# Supplementary material for: Worm orthologues of cytokinesis-associated proteins CIT and ASPM regulate neuronal microtubule dynamics and polarity in C. elegans
Source: PLoS Genet. 2026 Apr 15;22(4):e1012106. doi: 10.1371/journal.pgen.1012106 (PMC13108882; doi:10.1371/journal.pgen.1012106)
Supplement: S1 Appendix — (DOCX) [file pgen.1012106.s007.docx]

**Appendix-1**


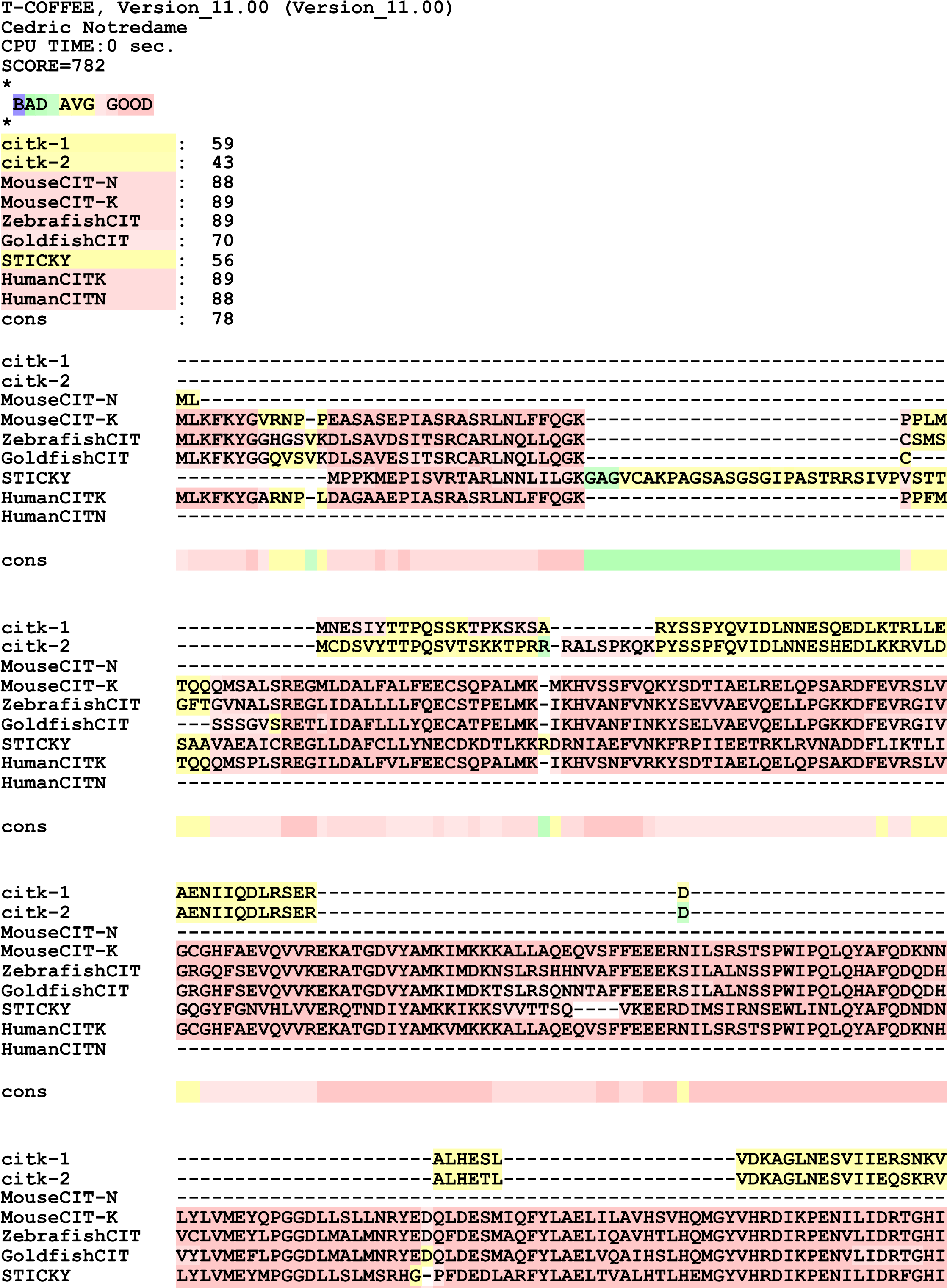


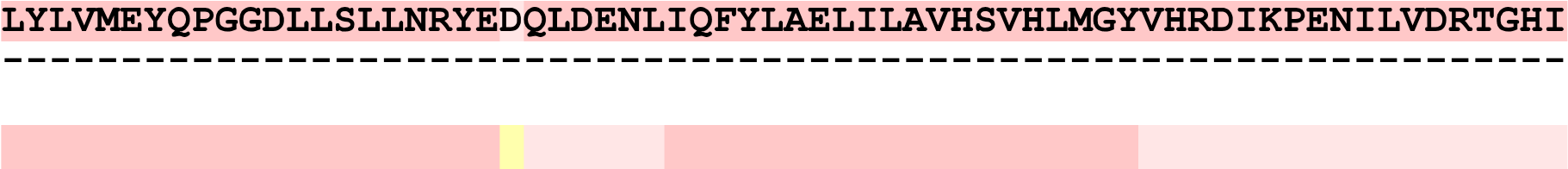


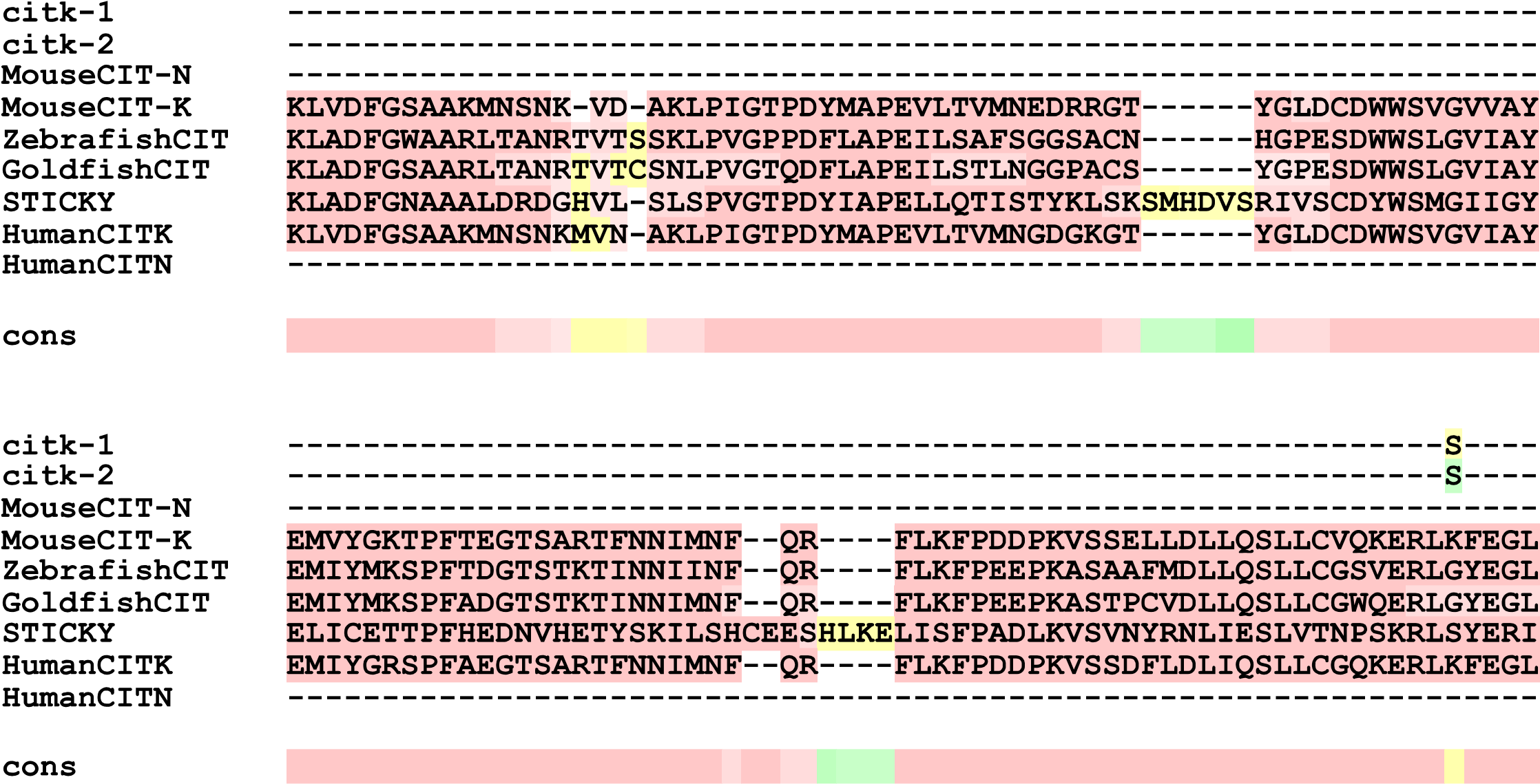


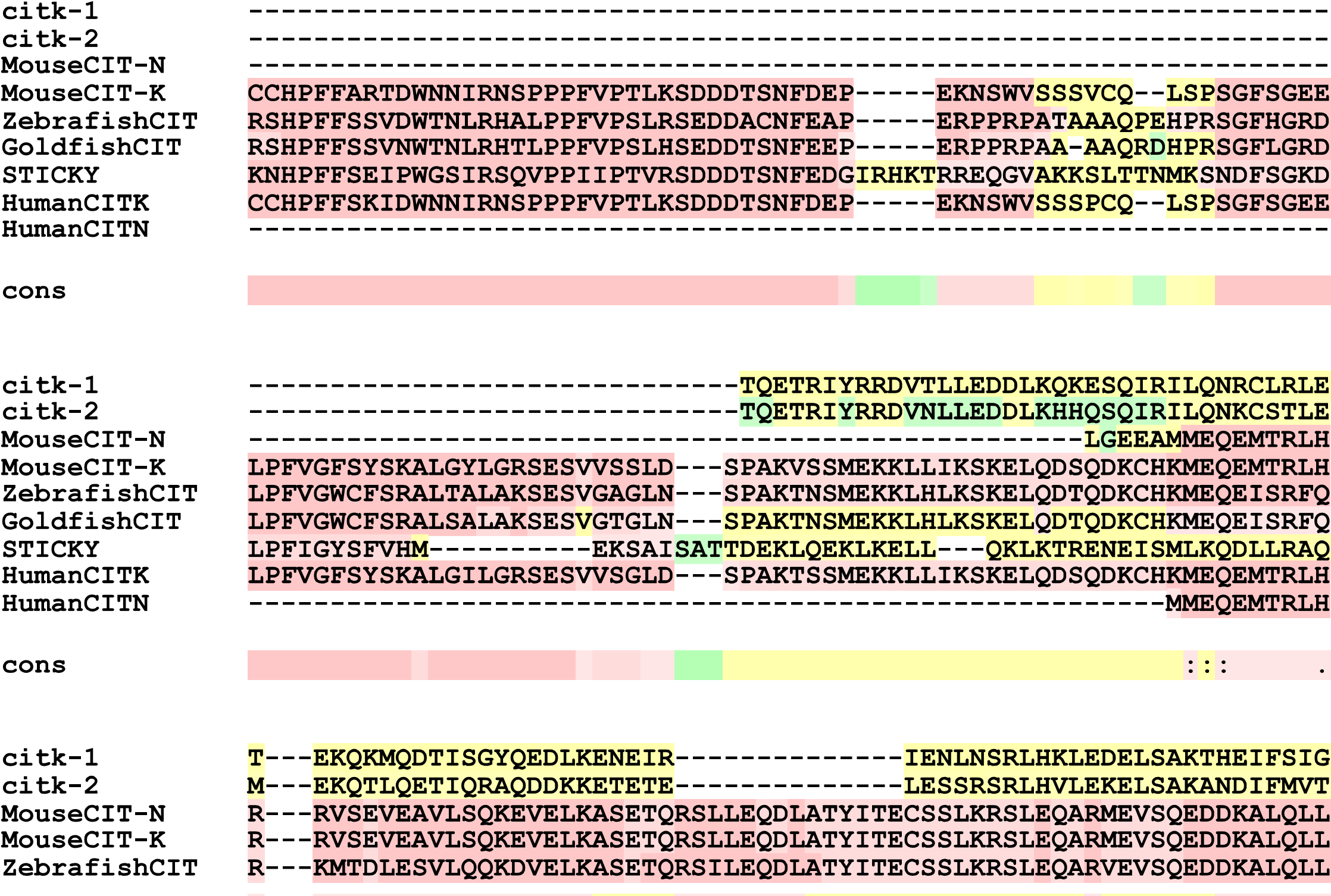


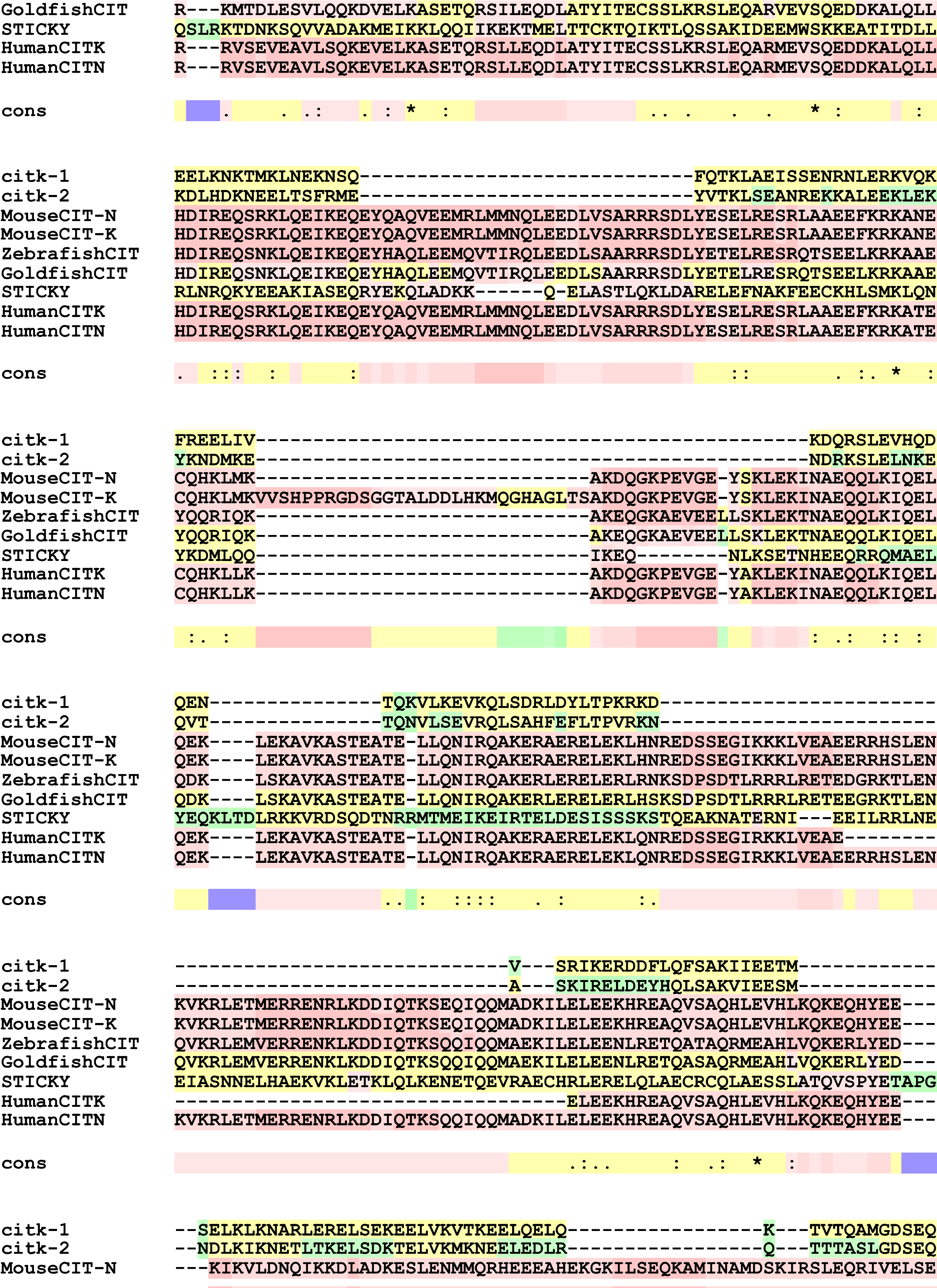


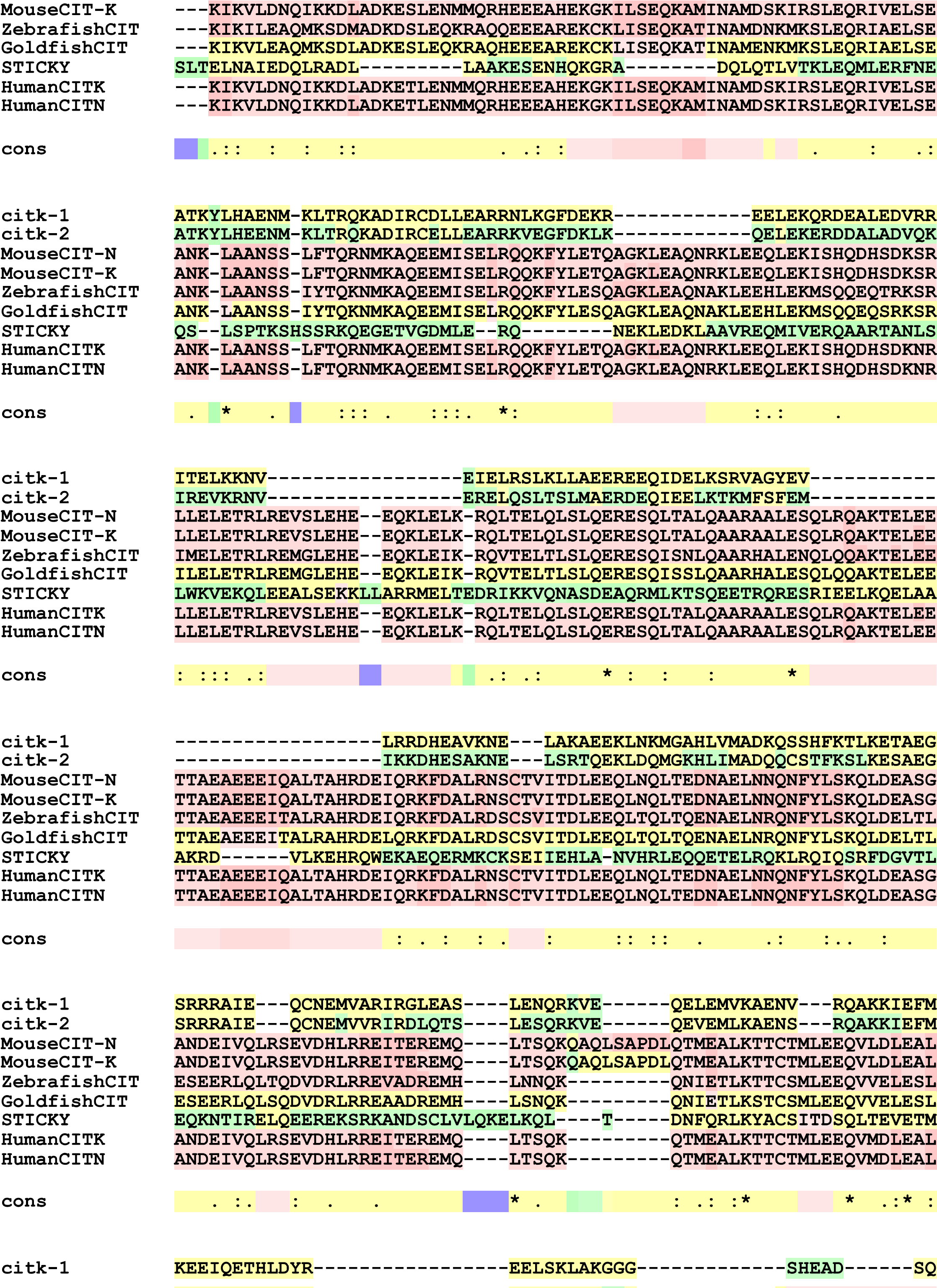


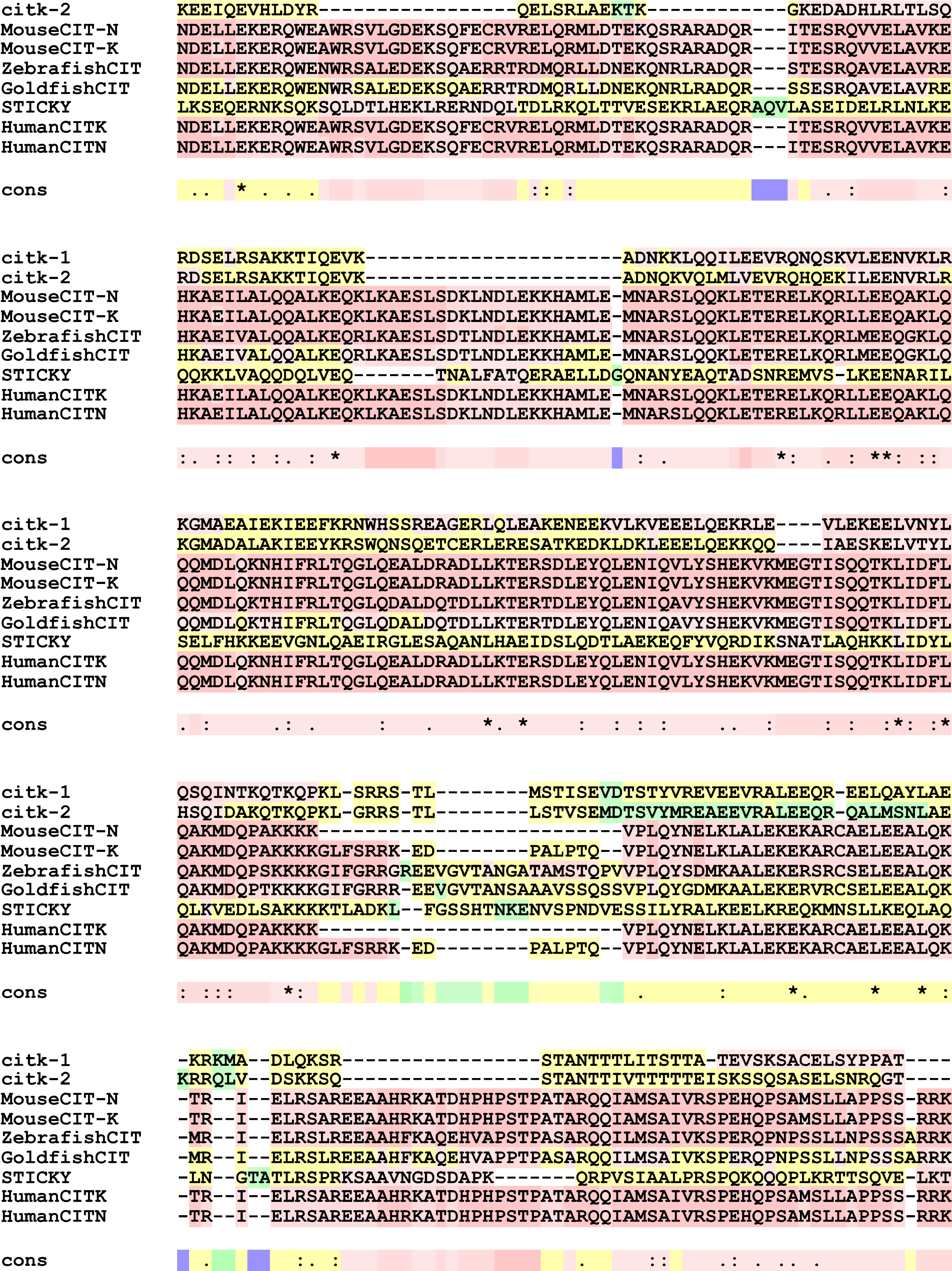


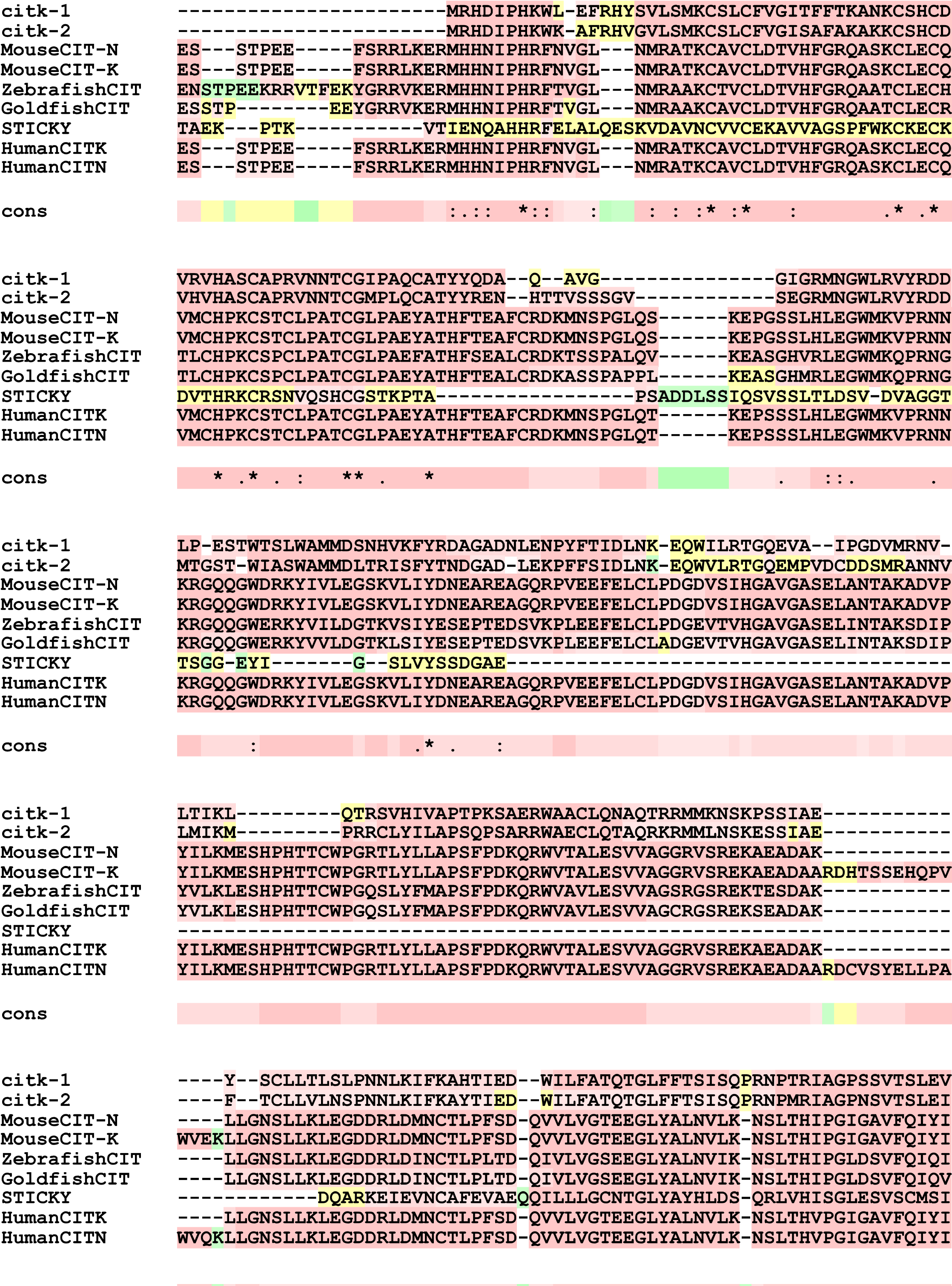


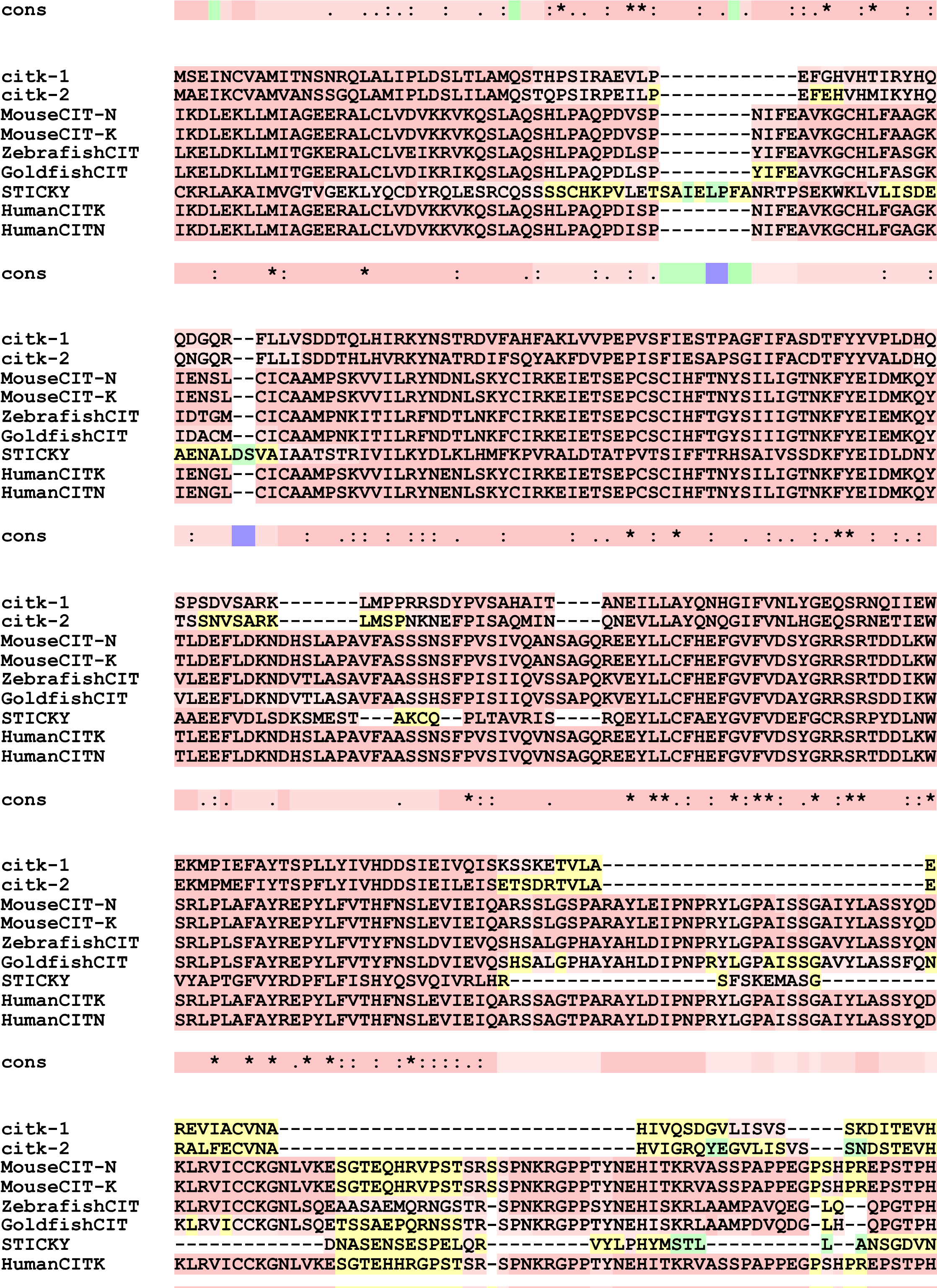


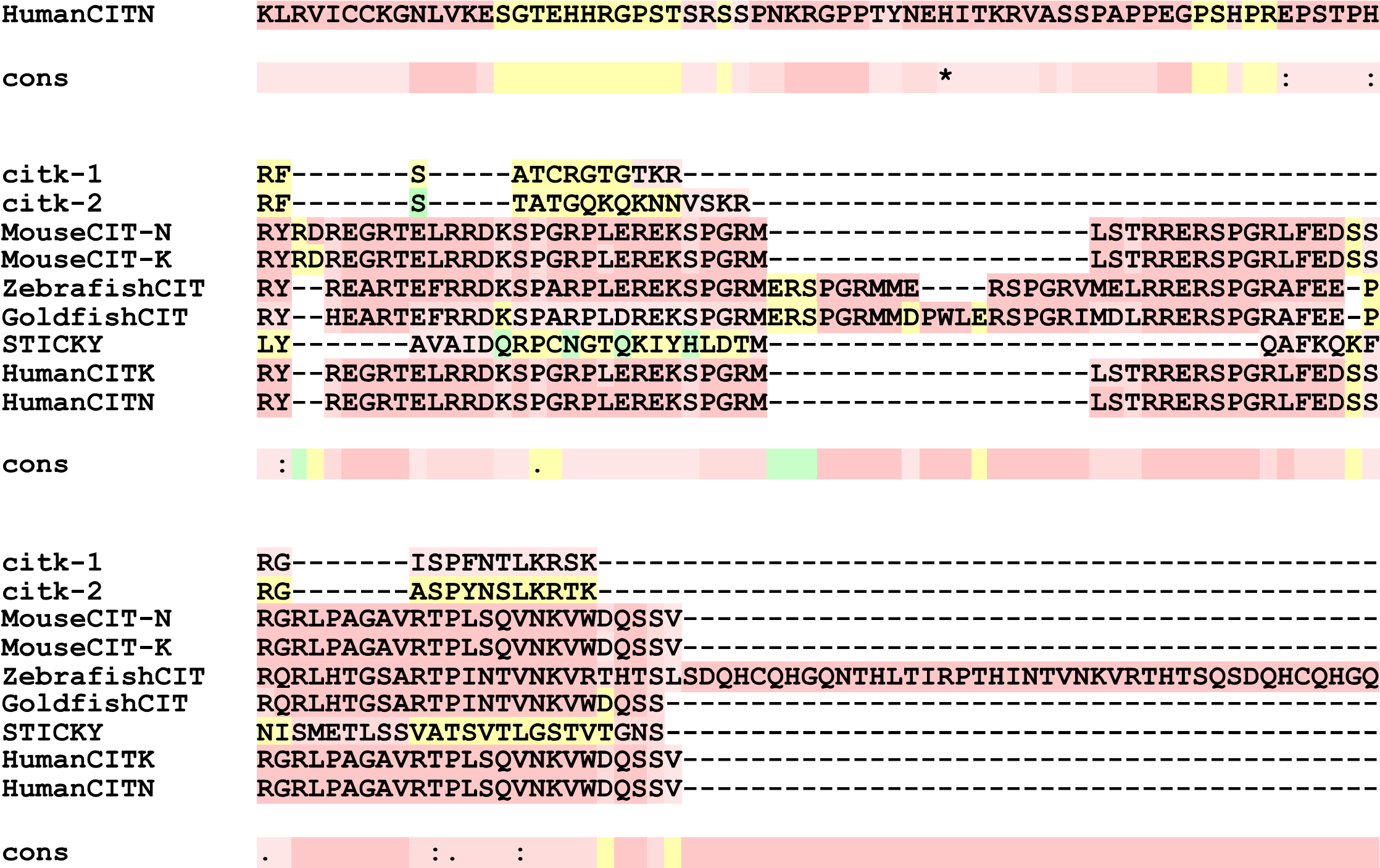


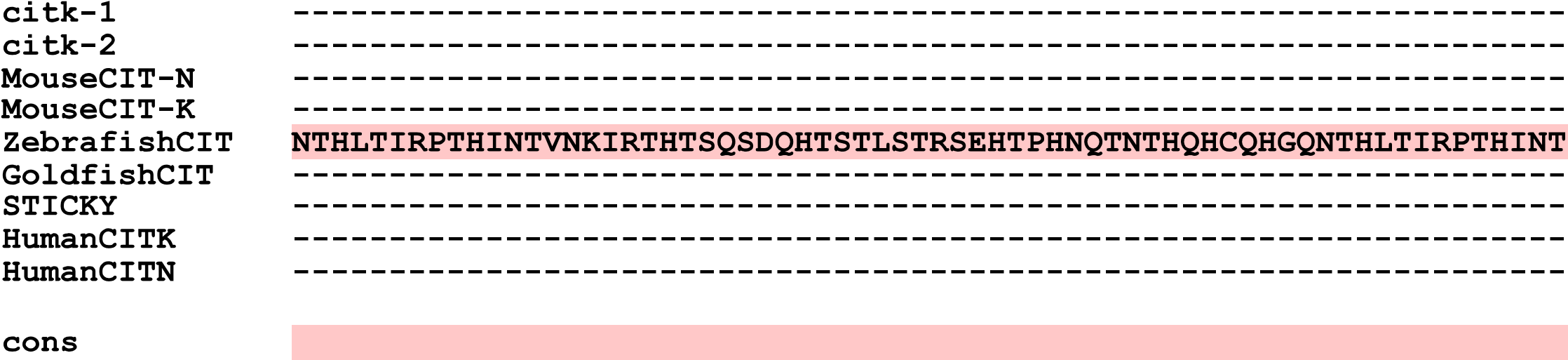


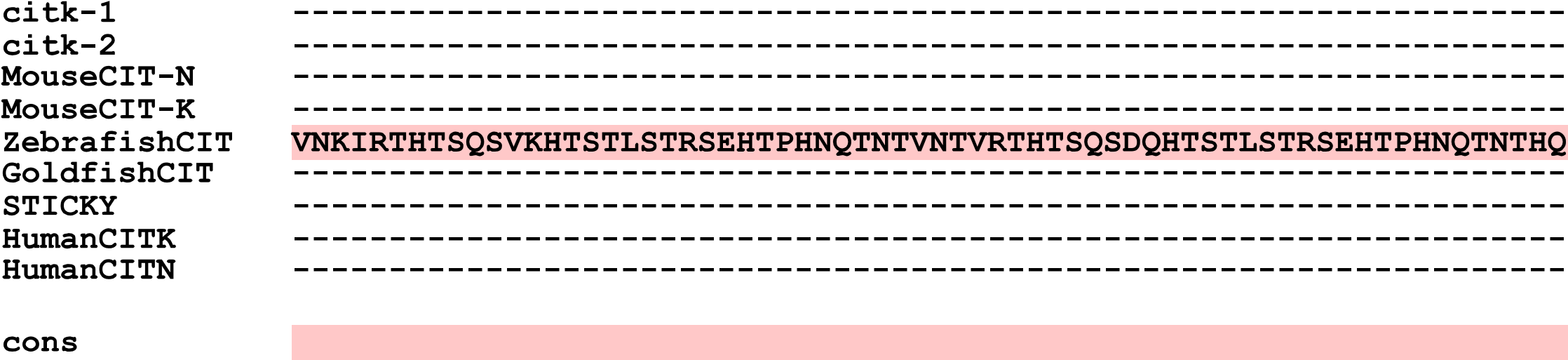


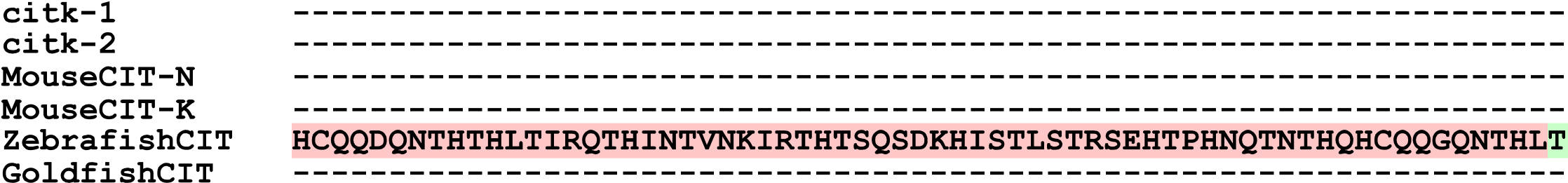


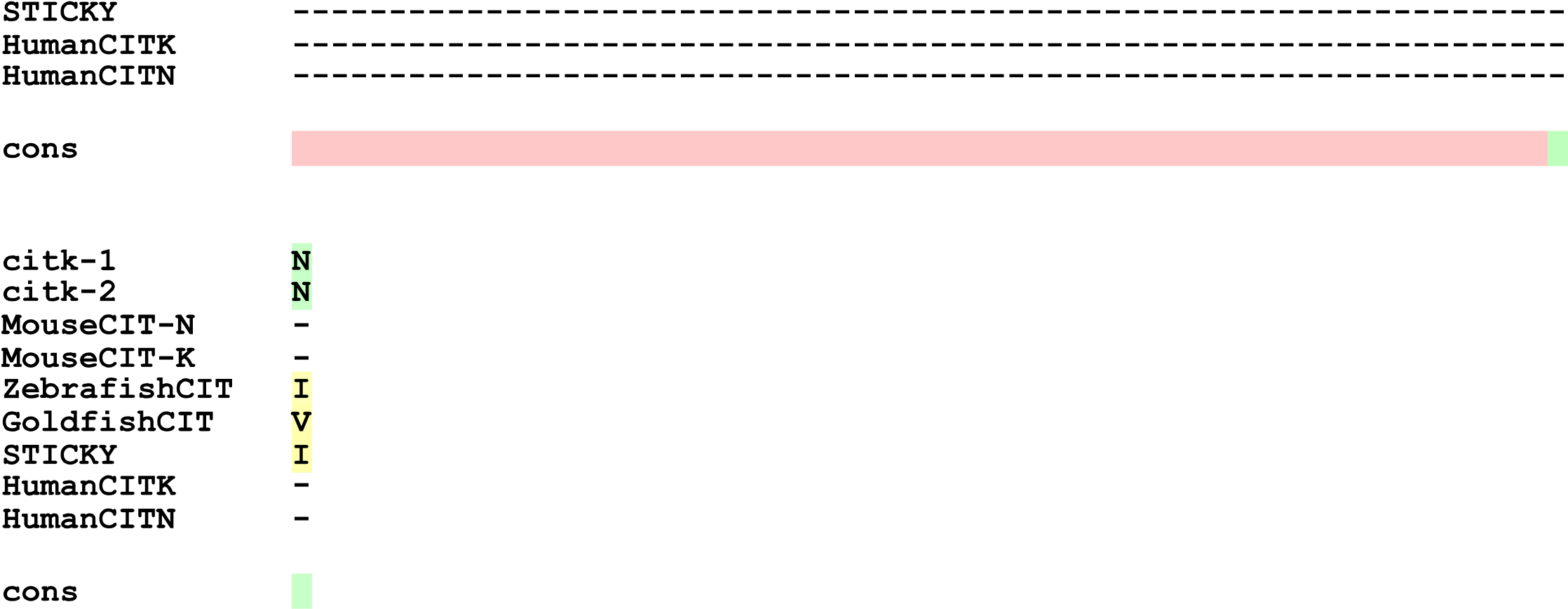


**Appendix 1**: **The sequence alignment of worm *citk-1* and *citk-2* with mammalian CIT proteins.**

The sequence alignment of *citk-1/W02B8.2* and *citk-2/F59A6.5* proteins with the long (N-terminal kinase) and short isoforms (CITN-neuronal isoform) of Human and mouse CIT proteins and CIT orthologs of zebrafish, goldfish and Drosophila (sticky). The color scheme is used to denote conserved residues. Alignment was generated by T-coffee (https://tcoffee.crg.eu/apps/tcoffee/do:mcoffee).
