# Supplementary figures and images for: Worm orthologues of cytokinesis-associated proteins CIT and ASPM regulate neuronal microtubule dynamics and polarity in C. elegans
Source: PLoS Genet. 2026 Apr 15;22(4):e1012106. doi: 10.1371/journal.pgen.1012106 (PMC13108882; doi:10.1371/journal.pgen.1012106)

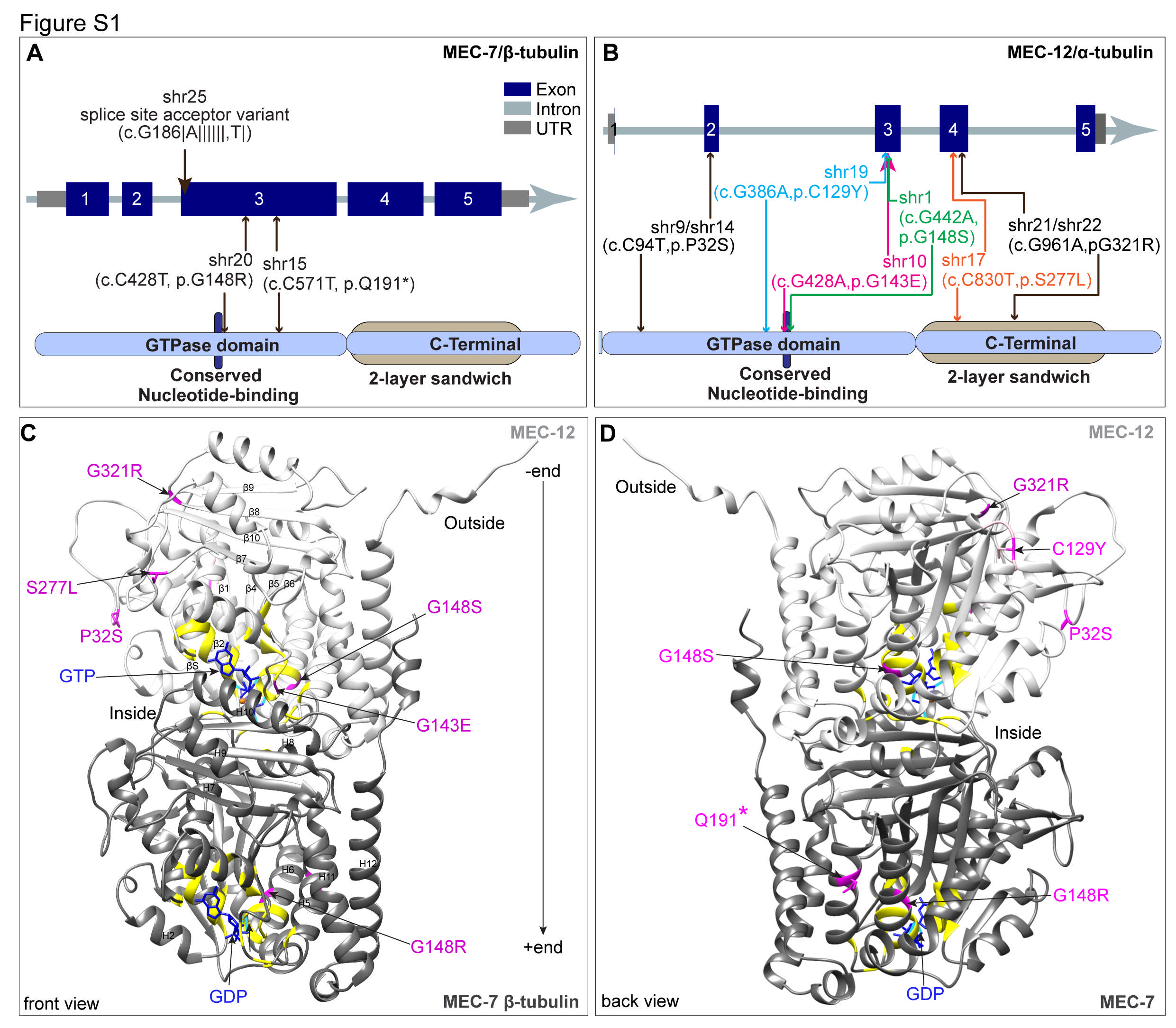

Supplement: S1 Fig — (A-B) Two-dimensional schematic illustration of the exon and intron of the mec-7 and mec-12 gene and protein structures with positions of identified candidate mutations labeled. The scale bar is in bp (base pairs). mec-7 and mec-12 gene. The mec-7(shr20) and mec-7(shr15) variants are in the nucleotide binding domain of the MEC-7 protein. The shr25 is a splice site acceptor variant at the junction of intron-2 and exon-3 which may influence RNA splicing and in effect MEC-7 protein folding or protein synthesis. The mec-12(shr9), mec-12(shr19), mec-12(shr10) and mec-12(shr1) variants are in the GTPase domain of protein and the mec-12(shr17), and mec-12(shr21) variants are in the C-terminal 2-layer sandwich domain (B). (C-D) Alphafold [112,113,114] generated three-dimensional schematics of a MEC-7 and MEC-12 tubulin dimer with locations of klp-7(0) suppressor variants highlighted in magenta using Chimera [115] and rotated to display front and back views of the dimer. As depicted in Fig C and D, the mec-7(shr15) mutation introduces a premature stop codon, a truncated protein if formed will lack the entire α-tubulin binding domain. The non-conservative mutations in the nucleotide binding pocket of protein like mec-7(G148R), mec-12(G148S) and mec-12(G143E) replace a simple flexible glycine amino acid with complex amino acids with positively (arginine) or negatively (glutamic acid) charged side chains or polar side chains with hydroxyl group (serine) might lead to steric hindrance and hence nucleotide binding and protein folding. The mec-12 mutation P32S is in the P loop and the G321R, S277L are present on the inter-dimer surface. These substitutions likely impact microtubule polymerization, nucleotide binding, or heterodimer interactions. The mec-12(C129Y) mutation replaces a small cysteine nucleotide capable of forming disulfide bonds with a large polar and aromatic tyrosine in interdimer surface. The mutation could disrupt the protein structure or stability or could even [file pgen.1012106.s008.tiff]

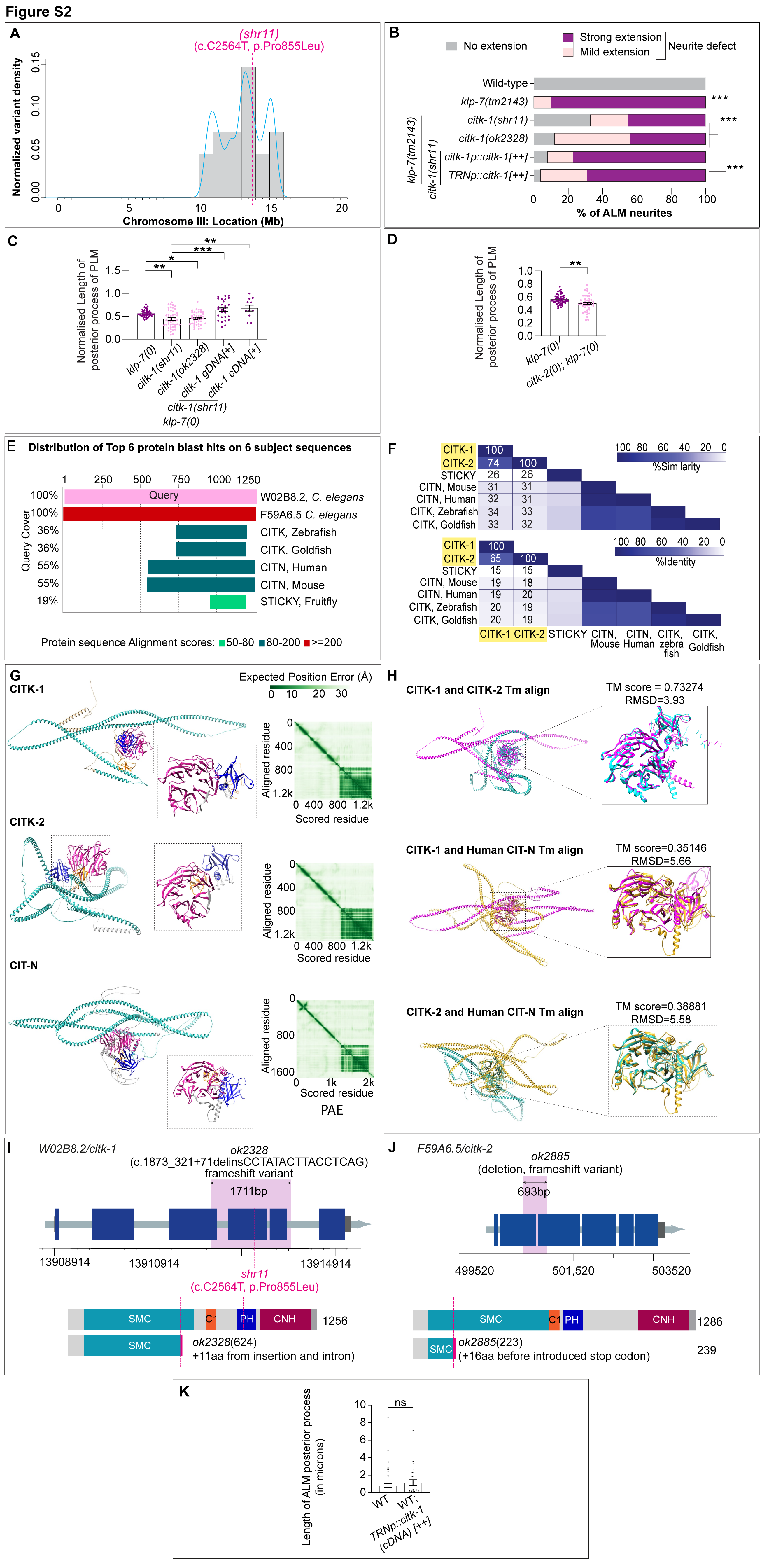

Supplement: S2 Fig — (A) Normalized variant density plot of Chromosome II obtained by EMS density mapping analysis of shr11 variant from the whole genome sequencing data using the method described in [110]. The variant density is normalized as the number of EMS induced variants per 500Mb (gray bars) along the chromosomal length in Mb. It peaks at the genomic position linked to shr11 variant. (B) Quantification of the percentage of ALM exhibiting ectopic extension phenotype in the wild-type, klp-7(0), klp-7(0); citk-1(shr11), klp-7(0); citk-1(shr11); citk-1(genomic DNA)[+] backgrounds, where citk-1 (genomic DNA) is the fosmid WRM062dD08. N = 3–5 independent replicates, n (number of neurons) = 45–60. P values from 2x2 Fischer’s exact test. (C-D) Quantification of the normalized length of PLM posterior process in (C) klp-7(0), citk-1(shr11); klp-7(0), citk-1(ok2328); klp-7(0), citk-1(shr11); klp-7(0); citk-1 genomic DNA [+] and citk-1(shr11); klp-7(0); citk-1 cDNA [+] where genomic citk-1 is the citk-1 fosmid WRM062dD08 and the citk-1(cDNA) is expressed under mechanosensory neuron specific promoter pmec-4 and in (D) klp-7(0) and citk-2(ok2885); klp-7(0) backgrounds. Normalized length of PLM posterior = (Absolute length/distance between the PLM cell body to the tip of the tail for the posterior neurite). For (D) N = 3–4 independent replicates, n (number of neurons) = 11–50. P values from Kruskal Wallis test followed by Dunn’s multiple comparisons. For (E) N = 3–4 independent replicates, n (number of neurons) = 42–45. (E) Graphical overview of the distribution of top blast hits [117] on the query sequence, W02B8.2/CITK-1, represented by the pink bar. The hits are shown aligned to the regions of query, below in color coded bars. (F) Heat map showing the percentage similarity and percentage identity shared between citron rho interacting kinase proteins from different species. (G) The Alphafold predicted structures of CITK-1, CITK-2, and the kinase-less isoform of mammalian CIT (CIT-N). The ins [file pgen.1012106.s009.tiff]

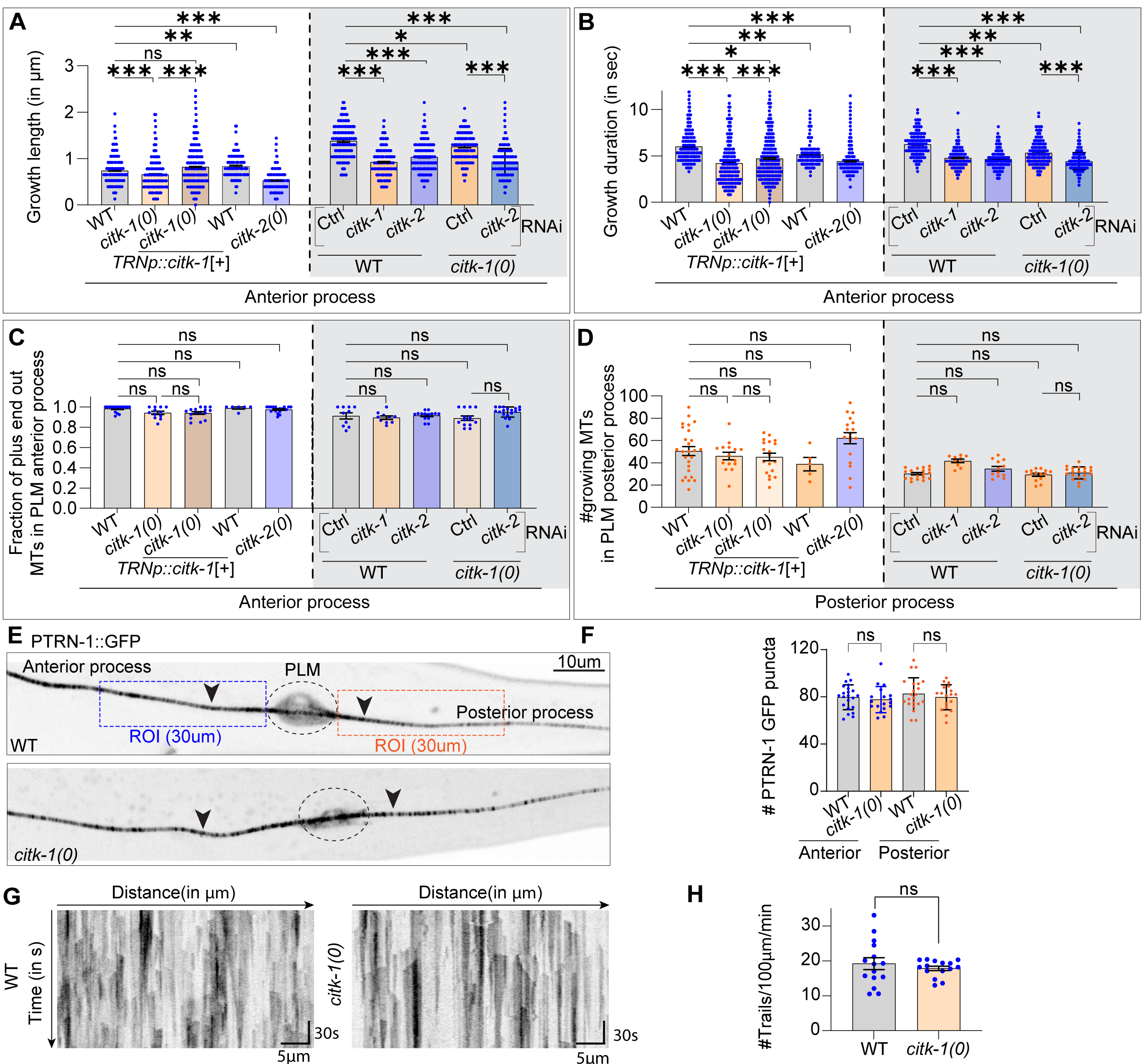

Supplement: S3 Fig — (A-B) Histograms depicting the growth length (A) and growth duration (B) quantified by determining the net pixel shift in the X and Y axis of kymographs obtained from PLM anterior processes of same backgrounds as Fig 3. N = 3–5 independent replicates, n (number of tracks) = 120–1081. (C) Quantification of fraction of plus-end-out microtubules (number of plus-end-out MTs/Total number of MTs) in the anterior process of given backgrounds. N = 3–5 independent replicates, n (number of tracks) = 6–18 (D) Quantification of number of growing microtubules per kymograph in the posterior process of PLM neurons for A-D, N = 3 independent replicates, n (number of neurons) = 6–26. (E) Representative confocal images of PLM neurons in L4 staged wild-type and citk-1(0) animals expressing transgene juEx6455(pmec-4::gfp::ptrn-1). Black arrowheads point to GFP::PTRN-1 puncta. The blue and orange dotted rectangle represent the 30µm ROI in the anterior and posterior process of PLM neuron. (F) Quantification of the number of GFP-PTRN-1 puncta seen in the 30µm ROIs in the anterior and posterior process of PLM neurons (as shown in Fig 3A and 3B). (G-H) Representative kymographs obtained by time lapse live imaging of PLM neurons expressing actin reporter, GFP::UtCH (G) and quantification of the actin trail density/100um/min in distal region of PLM neurons in WT and citk-1(0) animals (A-D, F, H) ***, P < 0.001; **, P < 0.01; *, P < 0.05, (A-D) P values from Kruskal Wallis test followed by Dunn’s multiple comparison test. The groups separated by dotted lines were analyzed independent of each other. (F) P values from ANOVA with Tukey’s multiple comparison test. (H) P values from Mann Whitney U Test. (A-D, F) Error bars represent SEM (Standard error mean), ns, not significant. (TIFF) [file pgen.1012106.s010.tiff]

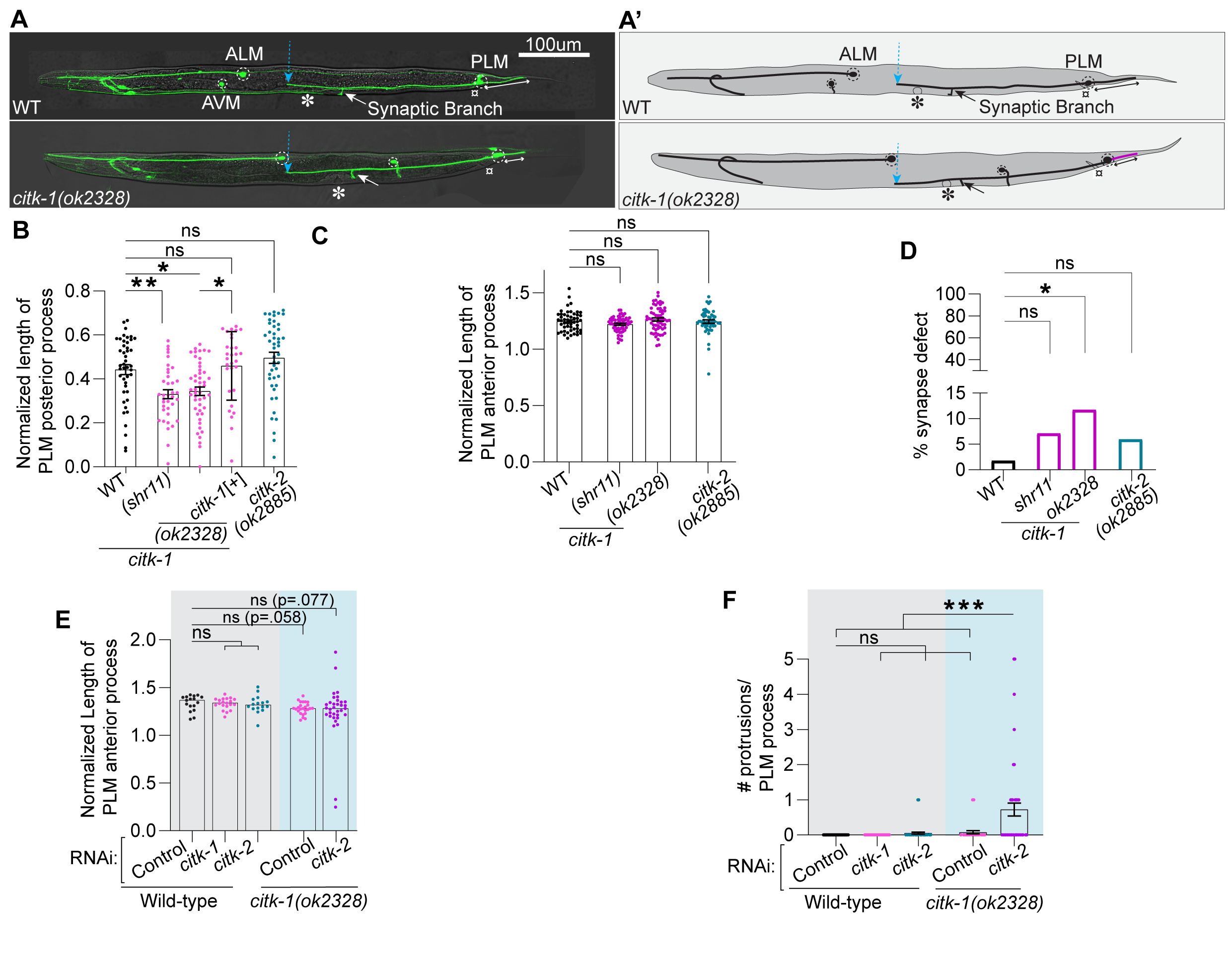

Supplement: S4 Fig — (A-A’) Representative confocal images and schematics of the ALM and PLM mechanosensory neurons in L4 stage wild-type and citk-1(ok2328) mutant animals. These neurons are labeled by muIs32(pmec-7::GFP) reporter. A dotted blue arrow marks the distal tip of PLM anterior process. The white arrow points to the ventral synaptic branch. White double-headed arrow is drawn along the length of PLM posterior process. Vulva on the ventral side of animal is marked by an asterisk (*), and anus is marked by a generic symbol (¤). (B-C) Quantification of the normalized length of PLM posterior process (B) and anterior process (C) in the wild-type, citk-1(shr11), citk-1(ok2328) and citk-2(ok2885) animals. (The PLM posterior process was also rescued by shrEx514(Pcitk-1::citk-1), extrachromosomal expression of a fosmid, WRM062dD08, containing complete genomic locus of citk-1, in citk-1(ok2328) mutant animals as quantified in (B). For B-C, N = 3–5 independent replicates, n (number of neurons) = 30–48. (D) Quantification of percentage of PLM neurons displaying a defect in the positioning or count of ventral synaptic branch in the wild-type, citk-1(shr11), citk-1(ok2328) and citk-2(ok2885) mutant animals. N = 3–5 independent replicates, n (number of neurons) = 51–68. (E) Quantification of the normalized length of PLM anterior process in the wild-type control and citk-1(0) RNAi sensitive background animals fed on bacteria expressing either empty vector (control) or citk-1 or citk-2 ds RNA for RNAi mediated knockdown. N = 3–5 independent replicates, n (number of neurons) = 17–34. (F) Quantification of number of short ectopic protrusions/ PLM neuron anterior process in the wild-type control and citk-1(0) RNAi sensitive background animals fed on bacteria expressing either empty vector (control) or citk-1 or citk-2 ds RNA for RNAi mediated knockdown. N = 3–5 independent replicates, n (number of neurons) = 32–56. Error bars represent SEM (Standard error mean), ns, not significant; ***, P < 0.001 [file pgen.1012106.s011.tif]

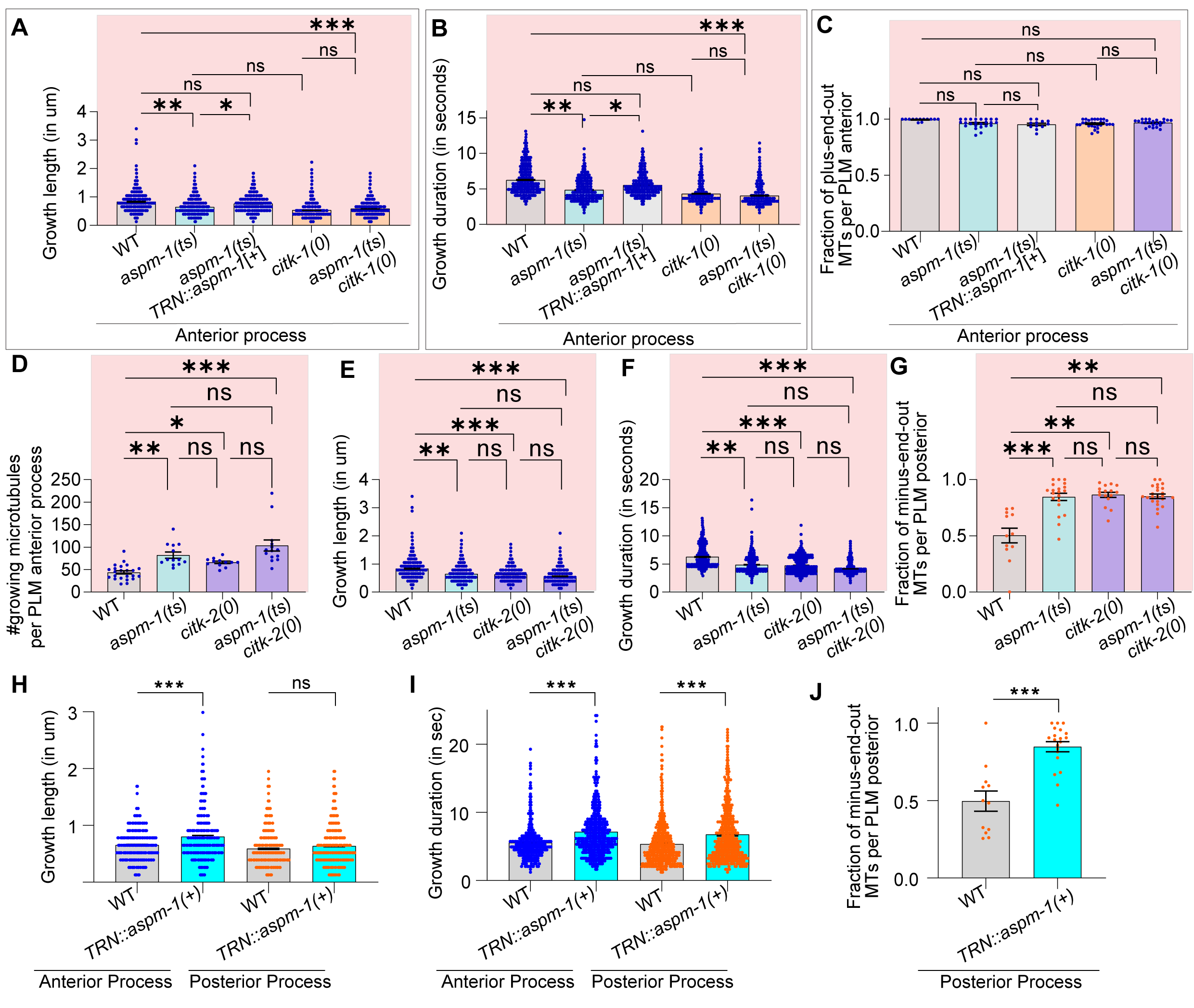

Supplement: S5 Fig — (A-B) Histograms depicting the growth length (A) and growth duration (B) of the EBP-2::GFP bound microtubules in the anterior process of PLM neurons in the wild-type and aspm-1(0) mutants, quantified by determining the net pixel shift in the X and Y axis (of kymographs) respectively. (C) Plot of fraction of plus-end out microtubules in PLM anterior process of respective genotypes. The wild-type, aspm-1(ts), aspm-1(ts); shrEx546(+), citk-1(0) and aspm-1(ts); citk-1(0) animals were grown at non-permissive temperature of 25°C. shrEx546 transgene expresses pmec-4::aspm-1. For A-C, N = 3–4 independent replicates, for A-B, n (number of tracks) = 112–756. P values from Kruskal Wallis test followed by Dunn’s multiple comparison and for C, n (number of PLM neurons) = 13–29. (D-G) Quantification of microtubule growth parameters (D-F) and fraction polarity (G) in PLM neurons of wild-type, aspm-1(ts), citk-2(ok2885) and aspm-1(ts); citk-2(ok2885) mutant backgrounds reared at non-permissive temperature of 25°C. For D-G, N = 3–4 independent replicates, for D, G, n (number of PLM neurons) = 12–23 and for E-F, n (number of tracks) = 512–1665. For D-G, P values from Kruskal Wallis test followed by Dunn’s multiple comparison. (H-J) Quantification of microtubule growth length(H), growth duration(I), and fraction polarity(J) in the anterior and posterior processes of WT and WT animals overexpressing aspm-1 as transgene shrEx530(pmec-4::aspm-1)(++). For H-J, N = 3 biological replicates, for H-I, n(no. of tracks) = 349–871 and for J, n(number of neurons) = 12–18. All animals in A-B and D-I express transgene juIs338(pmec-4::EBP-2::GFP). For H-J, P values from unpaired Student’s T-test. Error bars represent SEM (Standard error mean), ns, not significant; ***, P < 0.001; **, P < 0.01; *, P < 0.05. (TIFF) [file pgen.1012106.s012.tiff]

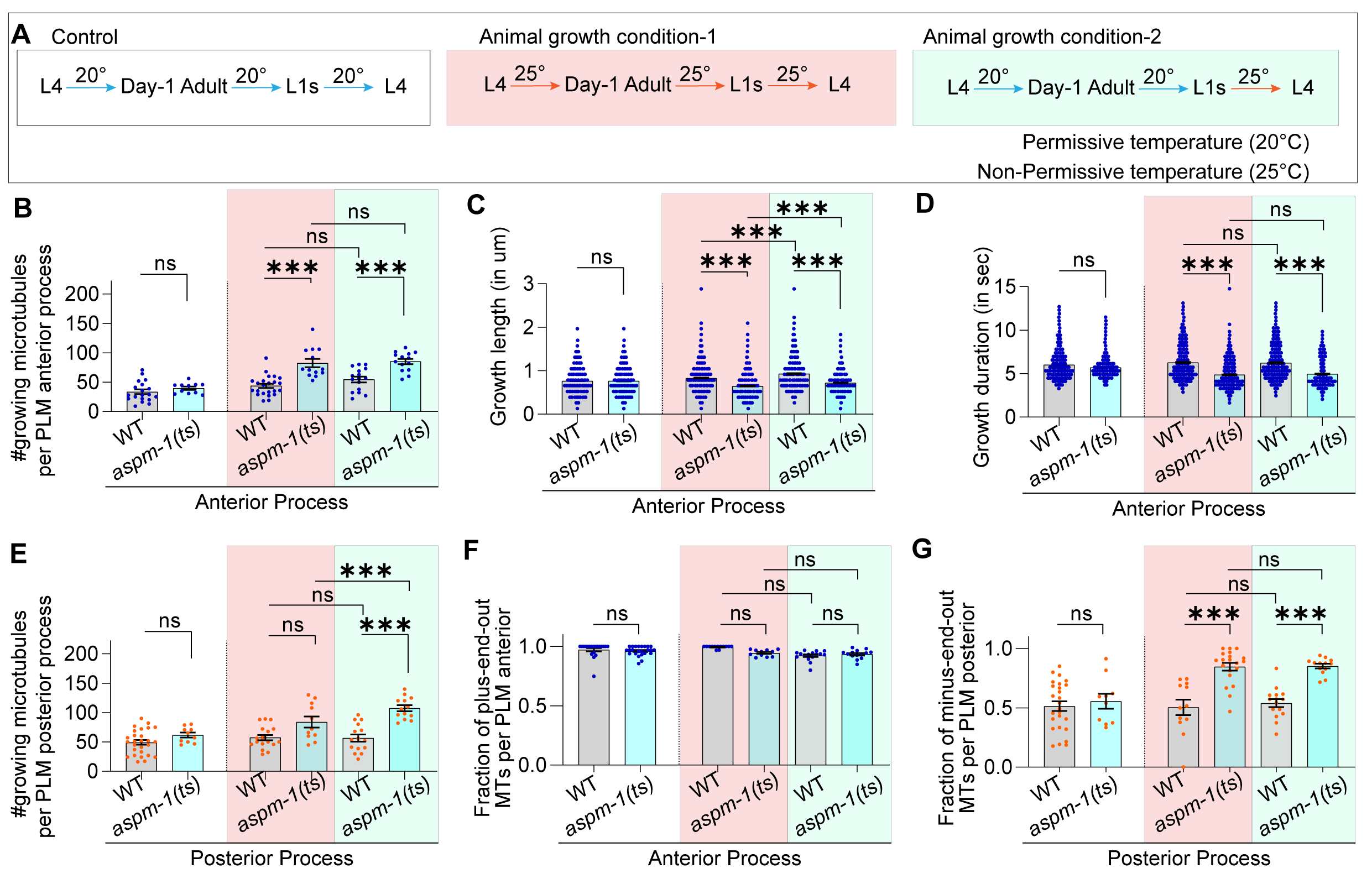

Supplement: S6 Fig — (A) Schematic illustrating control and test growth condition-1 and 2 animals were subjected-to for experiments in B-G. Animal growth condition-1 in pink illustrated rearing of animals at 25°C. Animal growth condition-2 in sky blue illustrates animals reared at 20°C. L1 progeny of these animals is transferred and grown to L4 stage at 25°C for experiments. (B-G) Quantification of microtubule growth parameters in PLM neurons of WT and aspm-1(ts), in control or test conditions. Kymographs were obtained from same ROI as described in Fig 3. The color of the background panel denotes the growth condition for respective datasets. For B-G, N = 3–4 biological replicates, For B, E-G, n(number of neurons) = 12–24, for C-D, n(number of tracks) = 805–1667. All animals in B-G express transgene juIs338(pmec-4::EBP-2::GFP). For B-G, Error bars represent SEM (Standard error mean), ns, not significant; ***, P < 0.001; **, P < 0.01; *, P < 0.05, P values from Kruskal Wallis test followed by Dunn’s multiple comparison. (TIFF) [file pgen.1012106.s013.tiff]

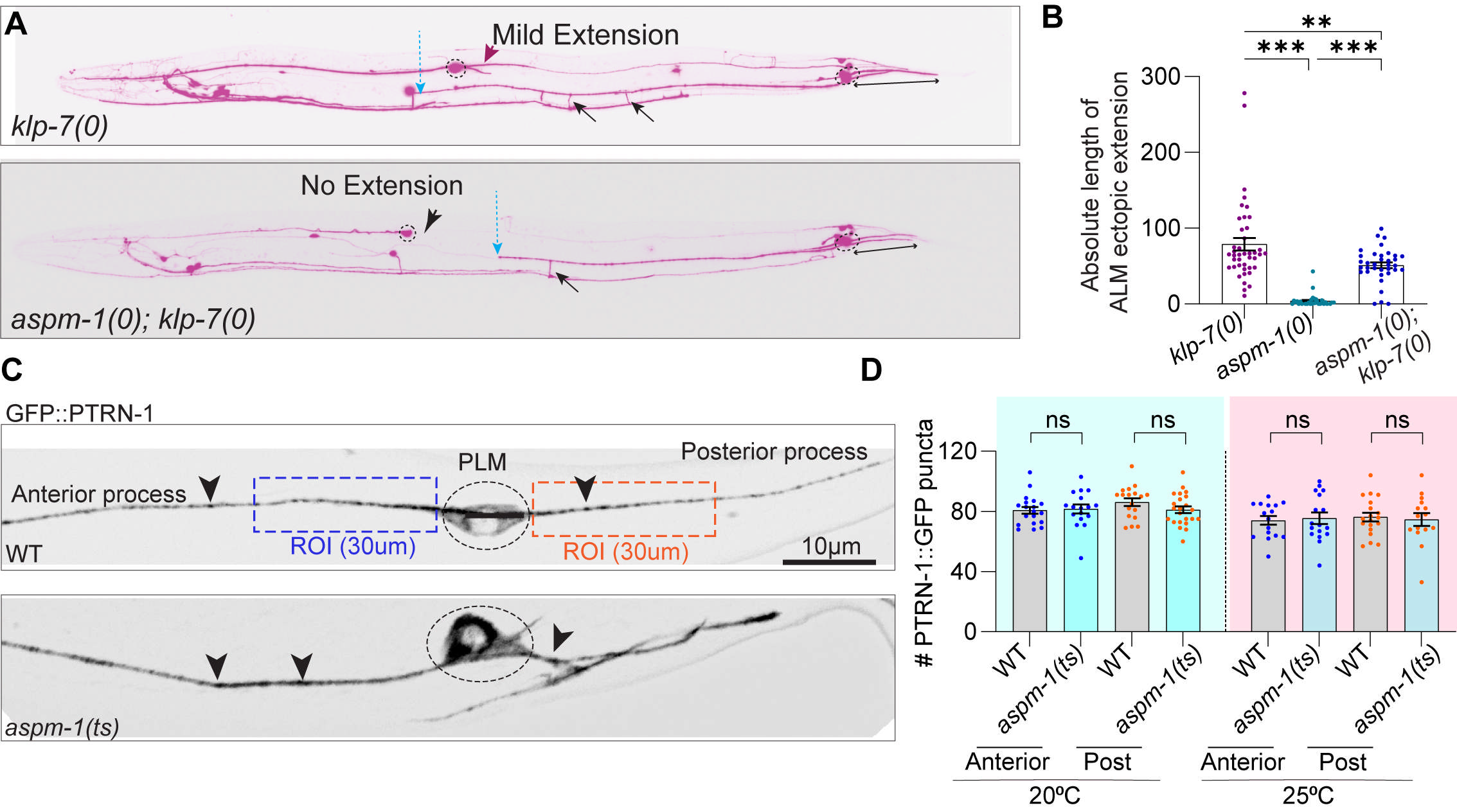

Supplement: S7 Fig — (A-B) Representative confocal images of ALM and PLM neurons in L4 staged klp-7(0) and aspm-1(0); klp-7(0) mutant animals grown at non-permissive temperature. The neurons are labeled by muIs32(pmec-7::GFP) reporter. However, the image has been inverted in ImageJ for presentation purposes. The ALM extends two mild posterior extensions (pink arrowhead) in klp-7(0) animal. The aspm-1(0); klp-7(0) double mutant shows no extension. The blue dotted arrow marks the distal tip of anterior process of PLM neuron, and the black double-headed arrow is drawn along the length of the PLM posterior process. Black solid arrows point to ventrally extended synaptic branches. (B) Quantification of the length of ALM posterior process in klp-7(0), aspm-1(ts) and aspm-1(ts); klp-7(0) mutants. N = 3 and n (number of neurons) = 21–46. (C) Representative confocal image showing distribution of PTRN-1::GFP puncta in the anterior and posterior process of PLM neurons in WT and aspm-1(ts) animals expressing transgene juEx6455(pmec-4::gfp::ptrn-1). The animals were reared at 25°C. The blue and orange dotted rectangle represent the 30µm ROI in the anterior and posterior process of PLM neuron. Black arrowheads point to GFP::PTRN-1 puncta. (D) Quantification of number of GFP::PTRN-1 puncta in WT and aspm-1(ts) grown at 20°C (in sky blue) and 25°C(in pink). The number of GFP::PTRN-1 puncta were quantified from 30µm ROI. N = 3 and n (number of neurons) = 15–23. For B, D Error bars represent SEM (Standard error mean). ***, P < 0.001; **, P < 0.01; * P < 0.05 and ns, not significant. For B, P values from Kruskal Wallis test followed by Dunn’s multiple comparison. For D, P values from ANOVA with Tukey’s multiple comparison test. (TIFF) [file pgen.1012106.s014.tiff]
