# Supplementary material for: Worm orthologues of cytokinesis-associated proteins CIT and ASPM regulate neuronal microtubule dynamics and polarity in C. elegans
Source: PLoS Genet. 2026 Apr 15;22(4):e1012106. doi: 10.1371/journal.pgen.1012106 (PMC13108882; doi:10.1371/journal.pgen.1012106)
Supplement: Attachment — Submitted filename: 2025_ PGENETICS-D-25-00512_Review.pdf [file pgen.1012106.s017.pdf]

Re: Manuscript PGENETICS-D-25-00512: "The cytokinesis associated proteins CITK and ASPM-1 regulate neuronal microtubule dynamics and polarity in *C. elegans*."

By conducting a screen for suppressors of neurite extension defects seen in mutants for the kinesin-like protein KLP-7, Sharma et al identified the "kinase-less" *C. elegans* homologs (CITK-1 and CITK-2) of the mammalian citron kinase as novel regulators of microtubule (MT) dynamics in mechanosensory neurons. Mouse neurons express two forms of the Citron protein: CIT-K, which includes the kinase domain, is highly-expressed in mitotic neuronal progenitors and its levels decrease in differentiated neurons, while CIT-N, which like the *C. elegans* proteins, lacks the kinase domain, is highly expressed in post-mitotic neurons and appears to play a role in regulating the actin cytoskeleton and organization of the Golgi apparatus.

Given that mammalian Abnormal Spindle-like Microcephaly-Associated Protein (ASPM) recruits CIT-K to the spindle, the authors also assessed the function of *C. elegans* ASPM-1 and found phenotypes like those seen in the *C. elegans citk-1* and *citk-2* mutants, leading them to propose that the CITK proteins and ASPM-1 function in the same pathway to regulate MT dynamics in mechanosensory neurons.

The experiments and results in this manuscript are well-presented, compelling, and identifying a role for CITK and ASPM-1 in post-mitotic neurons in *C. elegans* should be of general interest. However, my enthusiasm is tempered by some major issues (detailed below), which would have to be addressed to warrant publication.

<u>Major Issues</u>

a) The authors do not provide enough background about the mammalian Citron proteins (CIT-K and CIT-N) in the introduction, and they keep referring to the *C. elegans* proteins as kinases (sometimes, but not always, adding that they are "kinase-less"), which is confusing, as I kept wondering how their observations might be generally relevant when CITK-1 and CITK-2 do not have the kinase domain. I strongly recommend the authors add an introductory paragraph describing what is known about CIT-K and CIT-N in mammalian neurons, and that rather than referring to CITK-1 and CITK-2 as "citron kinases", they strictly refer to these two proteins as CIT-N homologs. If they make the importance of CIT-N clearer in the introduction (and consistently use "CIT-N", instead of "citron kinase" throughout), the work will be easier to follow and have a stronger impact.

b) Another major issue is that the authors conclude that ASPM-1 and CITK-1/2 function in the same pathway based on the similarity of phenotypes caused by loss of these proteins in mechanosensory neurons and on the fact that ASPM recruits CIT-K to the spindle in mammalian cells. However, it is not clear if ASPM only binds to CIT-K, or if it also binds to CIT-N. Regardless of what is known about ASPM binding to CIT-K and/or CIT-N, the conclusion that ASPM-1 and CITK-1/2 function in the same pathway in mechanosensory neurons is not fully supported by the data, as the authors do not test the hypothesis that these proteins might function in parallel. They should analyze ASPM-1 and CITK-1 and/or CITK-2 doubles (and/or triple) mutants, and assess if the phenotype is the same in these combinations or gets worse. Without this genetic interaction experiment, the authors cannot conclude that these proteins function in the same pathway.

c) The way that statistical results are presented in the figures makes is difficult to understand which datasets are being compared, so I couldn't reliably determine which differences were significant and which weren't. For example, in Fig. 2D and E, the comparison between *klp-7(0)* and *klp-7(0); citk-2(0)* doubles appear to not be significant in either case (p=0.177 in Fig. 2D, p=0.252 in Fig. 2E), but in the text the authors state that "A double mutant of *citk-2(ok2885); klp-7(0)* displayed significantly shorter ALM ectopic extension than the *klp-7(0)* mutant, suggesting a partial suppression of *klp-7(0)* phenotype (Figure 2A-A', D)", and they suggest a "trend" towards suppression of the posterior PLM outgrowth phenotype, which is difficult to ascertain from data presented (and the statistics, as shown do not suggest significance, so I wouldn't emphasize this result).

There are also some statistical results that do not make sense. For example, in Fig. 3F, the difference in fraction of plus vs minus-ended MTs in the Anterior PLM are noted as significant in some cases (e.g. p=0.051 for *citk-1* and p=.044 for *citk-2* mutants), but in the control RNAi experiment in *citk-1(0)* the difference in fraction of plus vs. minus-ended MTs is noted as not significant (p=0.45), even though the differences in bar heights and SEM seem virtually the same in all these conditions. Similarly, in the same panel the difference in fraction of plus vs minus-ended MTs in Posterior PLM is noted as not significant (p=0.319) in the *citk-2* mutant, but in *citk-1*

mutants the data again look almost the same as in *citk-2*, but in this case the difference is significant (p=0.015). I am just puzzled by the wide range of statistical results given the similar data sets.

Along the same lines, in Fig. 4B, RNAi against *citk-2* in *citk-1(0)* mutants shows p=0.114 when compared to control RNAi in *citk-1(0)*, but the authors state that "… shortening of posterior processes of PLM neurons, […] is synergistically further reduced on loss of *citk-2* in *citk-1(0)* mutant animals (Figure 4A, B)", which is not supported by the statistical results.

<u>Minor Issues</u>

a) I am not a big fan of the term "rein in" MTs, which the authors use multiple times. I don't know if this is an accepted term, but I would prefer something like "limits" instead.

b) For the rescue experiments in *klp-7(0)* backgrounds, what the authors are looking for is anti-suppression, meaning they are asking if the transgenes make the phenotype "worse" again in the *klp-7(0); citk-1(0)* background. Because of this, I would like to see that the transgenes themselves do not cause phenotypes in a wildtype background on their own, because if they do, it would be more difficult to interpret the anti-suppression/rescue data.

c) Line 145: "Using EMS density deep sequencing mapping …" a reference is needed, or a direction to readers to the Methods section, which has the appropriate references.

d) Fig. 2D needs a label for the Y-axis

e) Fig. 3G, H (and Fig. S3E, F): shouldn't values for wild-type controls be included to assess the degree of rescue relative to wild-type?

f) Lines 382-386: there are several references to "Fig. 3.3", which is not in the manuscript. Do the authors mean Fig. S4? Also, in this section the term "migration" is confusing. I think of migration as the process by which the cell body reaches its appropriate position. It seems like the authors are describing "guidance" or "outgrowth" instead of migration, and in fact, in some cases they use "guidance" and "migration" interchangeably. I suggest sticking to "guidance" or being more specific and calling it "neurite migration".

g) Line 414: "As one would expect …". Not sure what's meant here. Why would one expect that mispositioning the cell body would lead to neurite growth or guidance defects? If there's a reference demonstrating this, it should be added here.

h) Fig. 4: I think that a representative picture of a "wild-type" animal (really the *shrSi2; lin-15(-)* strain on control RNAi) is needed in 4A to compare to the two conditions shown. Also, the depth coding (which I think is erroneously called "temporal" in the figure legend, which would refer to time) is not useful here: the graded scale is not visible at this resolution, and the path along different depths in wild-type or mutants, and what we should glean from the way its shown, is not really described.

i) Fig. 5: the *aspm-1* mutation is referred to as a null *(0)* in the figure, but in the text it is referred to as a temperature sensitive *(ts)* mutation. The nomenclature should be consistent.

j) Fig. 6 is more of a summary of the results than an actual model. I envision that a model would suggest a pathway, or localization of the studied proteins relative to MTs. I just don't find this summary very useful, but it doesn't hurt to have it either.

k) In the discussion the authors bring up the actin cytoskeleton, and I found that a bit confusing. For example, in lines 568-572 a possible role for CIT-K in regulating actin dynamics is mentioned, but not elaborated upon, and the next sentence discusses CIT-K function in regulating astral MTs. So, I wasn't clear as to why the work on actin was mentioned. Similarly, in lines 586-592, the authors seem to bring up the possibility that a CITK-MyosinII-RhoA complex may "bridge" actin and MTs in neurons, as they do in cytokinesis. However, given that the authors did not examine actin in this work (nor do they provide examples of how actin regulates neurite outgrowth from other work), this speculation seems out of place. In the following

sentence (lines 592-594) the authors state that they found PLM positioning and guidance defects in the *citk* mutants, but it's not clear how this fits in a section discussing actin, and/or actin-MT cross-bridging proteins like Kif21b. Do mutations in actin regulators also cause PLM positioning and guidance defects? Defects found with loss of a cross-bridging protein, like Kif21b, do not provide strong support for a role for actin, as it would be necessary to functionally separate actin vs. MT-regulating activities for such a protein.
