# Supplementary material for: Worm orthologues of cytokinesis-associated proteins CIT and ASPM regulate neuronal microtubule dynamics and polarity in C. elegans
Source: PLoS Genet. 2026 Apr 15;22(4):e1012106. doi: 10.1371/journal.pgen.1012106 (PMC13108882; doi:10.1371/journal.pgen.1012106)
Supplement: Attachment — Submitted filename: Response to reviewers 270126.docx [file pgen.1012106.s019.docx]

PGENETICS-D-25-00512
The cytokinesis-associated proteins CITK and ASPM-1 regulate neuronal microtubule dynamics and polarity in C. elegans.
PLOS Genetics

Dear Dr. Ghosh-Roy,

Thank you for submitting your manuscript to PLOS Genetics. After careful consideration, we feel that it has merit but does not fully meet PLOS Genetics's publication criteria as it currently stands. In particular, reviewers raised questions about whether ASPM-1 and CITK-1/2 function in the same pathway (suggesting genetic tests to examine this); whether the worm proteins examined were true paralogs of the human proteins; and had concerns with the clarity of the writing. We invite you to submit a revised version of the manuscript that addresses the points raised during the review process.

Please submit your revised manuscript within 60 days Aug 12 2025 11:59PM. If you will need more time than this to complete your revisions, please reply to this message or contact the journal office at [plosgenetics@plos.org](mailto:plosgenetics@plos.org). When you're ready to submit your revision, log on to <https://www.editorialmanager.com/pgenetics/> and select the 'Submissions Needing Revision' folder to locate your manuscript file.

Please include the following items when submitting your revised manuscript:

* A rebuttal letter that responds to each point raised by the editor and reviewer(s). You should upload this letter as a separate file labeled 'Response to Reviewers'. This file does not need to include responses to any formatting updates and technical items listed in the 'Journal Requirements' section below.
* A marked-up copy of your manuscript that highlights changes made to the original version. You should upload this as a separate file labeled 'Revised Manuscript with Track Changes'.
* An unmarked version of your revised paper without tracked changes. You should upload this as a separate file labeled 'Manuscript'.

If you would like to make changes to your financial disclosure, competing interests statement, or data availability statement, please make these updates within the submission form at the time of resubmission. Guidelines for resubmitting your figure files are available below the reviewer comments at the end of this letter.

We look forward to receiving your revised manuscript.

Kind regards,

Jeremy Nance
Academic Editor
PLOS Genetics

Pablo Wappner
Section Editor
PLOS Genetics

Aimée Dudley
Editor-in-Chief
PLOS Genetics

Anne Goriely
Editor-in-Chief
PLOS Genetics

**Journal Requirements:**

1) Please ensure that the CRediT author contributions listed for every co-author are completed accurately and in full.
At this stage, the following Authors/Authors require contributions: Sunanda Sharma, Keerthana Ponniah, and Anindya Ghosh-Roy. Please ensure that the full contributions of each author are acknowledged in the "Add/Edit/Remove Authors" section of our submission form.
The list of CRediT author contributions may be found here: <https://journals.plos.org/plosgenetics/s/authorship#loc-author-contributions>

2) Please provide an Author Summary. This should appear in your manuscript between the Abstract (if applicable) and the Introduction, and should be 150-200 words long. The aim should be to make your findings accessible to a wide audience that includes both scientists and non-scientists. Sample summaries can be found on our website under Submission Guidelines:
<https://journals.plos.org/plosgenetics/s/submission-guidelines#loc-parts-of-a-submission>

3) Please upload all main figures as separate Figure files in .tif or .eps format. For more information about how to convert and format your figure files please see our guidelines: 
<https://journals.plos.org/plosgenetics/s/figures>

4) We have noticed that you have uploaded Supporting Information files, but you have not included a list of legends. Please add a full list of legends for your Supporting Information files after the references list.

5) Please ensure that the funders and grant numbers match between the Financial Disclosure field and the Funding Information tab in your submission form. Note that the funders must be provided in the same order in both places as well. State what role the funders took in the study. If the funders had no role in your study, please state: "The funders had no role in study design, data collection and analysis, decision to publish, or preparation of the manuscript.".
If you did not receive any funding for this study, please simply state: u201cThe authors received no specific funding for this work.u201d

**Reviewers' comments:**

Reviewer's Responses to Questions

**Comments to the Authors:
Please note here if the review is uploaded as an attachment.**

Reviewer #1: Review is uploaded as an attachment

By conducting a screen for suppressors of neurite extension defects seen in mutants for the kinesin-like protein KLP-7, Sharma et al identified the “kinase-less” *C. elegans* homologs (CITK-1 and CITK-2) of the mammalian citron kinase as novel regulators of microtubule (MT) dynamics in mechanosensory neurons. Mouse neurons express two forms of the Citron protein: CIT-K, which includes the kinase domain, is highly-expressed in mitotic neuronal progenitors and its levels decrease in differentiated neurons, while CIT-N, which like the *C. elegans* proteins, lacks the kinase domain, is highly expressed in post-mitotic neurons and appears to play a role in regulating the actin cytoskeleton and organization of the Golgi apparatus.

Given that mammalian Abnormal Spindle-like Microcephaly-Associated Protein (ASPM) recruits CIT-K to the spindle, the authors also assessed the function of *C. elegans* ASPM-1 and found phenotypes like those seen in the *C. elegans citk-1* and *citk-2* mutants, leading them to propose that the CITK proteins and ASPM-1 function in the same pathway to regulate MT dynamics in mechanosensory neurons.

The experiments and results in this manuscript are well-presented, compelling, and identifying a role for CITK and ASPM-1 in post-mitotic neurons in *C. elegans* should be of general interest. However, my enthusiasm is tempered by some major issues (detailed below), which would have to be addressed to warrant publication.

Major Issues

a) The authors do not provide enough background about the mammalian Citron proteins (CIT-K and CIT-N) in the introduction, and they keep referring to the *C. elegans* proteins as kinases (sometimes, but not always, adding that they are “kinase-less”), which is confusing, as I kept wondering how their observations might be generally relevant when CITK-1 and CITK-2 do not have the kinase domain. I strongly recommend the authors add an introductory paragraph describing what is known about CIT-K and CIT-N in mammalian neurons, and that rather than referring to CITK-1 and CITK-2 as “citron kinases”, they strictly refer to these two proteins as CIT-N homologs. If they make the importance of CIT-N clearer in the introduction (and consistently use “CITN”, instead of “citron kinase” throughout), the work will be easier to follow and have a stronger impact.

Response: Please see lines 126-141. We have added the following paragraph in the introduction to shed light on the role of CIT proteins in neurons:

Mammalian *Cit* gene majorly encodes two isoforms: a long isoform CIT-K, which has an N-terminal kinase domain and is ubiquitously expressed in proliferating cells, and a short isoform, CIT-N, which lacks the N-terminal kinase domain and is expressed exclusively in the post-mitotic and differentiated cells of the central nervous system (52, 53).

It has been shown that the longer isoform CITK orchestrates midbody maturation and organization of spindle microtubule arrays in dividing cells (50, 52, 54-58). Furthermore, in-situ hybridization experiments, using the kinase domain, showed localization of CITK protein in a specific neuronal population in the cortex and thalamus, suggesting a role in neuronal differentiation (59). The neuronal cell lines studies suggest the involvement of CITK as well as CITN, the kinase-less isoform of CIT, in limiting neurite outgrowth (59-61). Additionally, *in-vitro* cultures and *in vivo* knockdown studies show that CIT-N regulates dendritic spine density and maturation. Notably, CIT-N is shown to be localized with spine-associated Golgi compartments and post-synaptic densities *in vitro* (59, 61-63). However, the roles of CIT proteins in the regulation of microtubule dynamics in post-mitotic neurons have not been explored.

b) Another major issue is that the authors conclude that ASPM-1 and CITK-1/2 function in the same pathway based on the similarity of phenotypes caused by loss of these proteins in mechanosensory neurons and on the fact that ASPM recruits CIT-K to the spindle in mammalian cells. However, it is not clear if ASPM only binds to CIT-K, or if it also binds to CIT-N. Regardless of what is known about ASPM binding to CIT-K and/or CIT-N, the conclusion that ASPM-1 and CITK-1/2 function in the same pathway in mechanosensory neurons is not fully supported by the data, as the authors do not test the hypothesis that these proteins might function in parallel. They should analyze ASPM-1 and CITK-1 and/or CITK-2 doubles (and/or triple) mutants and assess if the phenotype is the same in these combinations or gets worse. Without this genetic interaction experiment, the authors cannot conclude that these proteins function in the same pathway.

Response: Thanks for this interesting suggestion. We examined the microtubule dynamics in both *aspm-1(ts); citk-1(0)* and *aspm-1(ts); citk-2(0)* double mutant animals. We observed no synergistic effects on microtubule dynamics of the double mutant animals. Please see Figure 5 and Figure S5 and lines 405-409. Since the *citk-1(0)* and *citk-2(0)* double mutants are lethal, and *aspm-1(or645)* is a ts allele, we could not test triple mutants.

The way that statistical results are presented in the figures makes is difficult to understand which datasets are being compared, so I couldn’t reliably determine which differences were significant and which weren’t. For example, in Fig. 2D and E, the comparison between *klp-7(0)* and *klp-7(0); citk-2(0)* doubles appear to not be significant in either case (p=0.177 in Fig. 2D, p=0.252 in Fig. 2E), but in the text the authors state that “A double mutant of *citk-2(ok2885); klp-7(0)* displayed significantly shorter ALM ectopic extension than the *klp-7(0)* mutant, suggesting a partial suppression of *klp-7(0)* phenotype (Figure 2A-A’, D)”, and they suggest a “trend” towards suppression of the posterior PLM outgrowth phenotype, which is difficult to ascertain from data presented (and the statistics, as shown do not suggest significance, so I wouldn’t emphasize this result).

Response: We have revised the statistics and data presentation for simplicity and clear visualization. We have separated *citk-2* from *citk-1,* and as the figure suggests, *citk-2(ok2885)* mutation suppresses the *klp-7(0)* ALM posterior extension and PLM overgrowth phenotype, which is statistically significant. Please see Figure 2 and Figure S2.

There are also some statistical results that do not make sense. For example, in Fig. 3F, the difference in fraction of plus vs minus-ended MTs in the Anterior PLM are noted as significant in some cases (e.g. p=0.051 for *citk-1* and p=.044 for *citk-2* mutants), but in the control RNAi experiment in *citk-1(0)* the difference in fraction of plus vs. minus-ended MTs is noted as not significant (p=0.45), even though the differences in bar heights and SEM seem virtually the same in all these conditions. Similarly, in the same panel the difference in fraction of plus vs minus-ended MTs in Posterior PLM is noted as not significant (p=0.319) in the *citk-2* mutant, but in *citk-1* mutants the data again look almost the same as in *citk-2*, but in this case the difference is significant (p=0.015). I am just puzzled by the wide range of statistical results given the similar data sets.

Response: For clear visualization of differences and to simplify data presentation, we have separated the data for anterior and posterior processes. In the graph for the fractional polarity of microtubules in the posterior process, we have represented the fraction of minus-end-out microtubules (number of minus-end-out microtubules/Total number of microtubules), since we are observing a change in minus-end-out microtubule population in the posterior process of mutants. Please see revised Figure 3D.

The anterior process of the PLM neuron displays a plus-end-out microtubule polarity. We did not observe any change in the MT polarity in the PLM anterior process, as depicted in Figure S3C, where we have plotted the fraction of plus-end-out microtubule population (number of plus-end-out microtubules/Total number of microtubules).

Please note that the sample sizes for some of the cases were smaller, leading to wide variation in the statistical results. We have increased the sample size and separated the datasets for clarity and statistical comparison.

Along the same lines, in Fig. 4B, RNAi against *citk-2* in *citk-1(0)* mutants shows p=0.114 when compared to control RNAi in *citk-1(0)*, but the authors state that “… shortening of posterior processes of PLM neurons, […] is synergistically further reduced on loss of *citk-2* in *citk-1(0)* mutant animals (Figure 4A, B)”, which is not supported by the statistical results.

Response: Thanks for pointing this out. We have increased the sample size and reanalyzed the data. The synergistic loss shows one-star significance. Please see Figure 4B.

Minor Issues

a) I am not a big fan of the term “rein in” MTs, which the authors use multiple times. I don’t know if this is an accepted term, but I would prefer something like “limits” instead.

Response: Thanks for the suggestion. Please note that the text has been edited. See line 143, 322, 541.

b) For the rescue experiments in *klp-7(0)* backgrounds, what the authors are looking for is anti-suppression, meaning they are asking if the transgenes make the phenotype “worse” again in the *klp-7(0); citk-1(0)* background. Because of this, I would like to see that the transgenes themselves do not cause phenotypes in a wild-type background on their own, because if they do, it would be more difficult to interpret the antisuppression/rescue data.

Response: The overexpression of *Pmec-4::citk-1(cDNA)* transgene in the wild-type animals at the same concentration as what was used for rescue experiments showed no significant change in the morphology of PLM neurons. The quantification of the posterior process of ALM neurons is plotted, and it shows no significant difference, as shown below. Please see Fig S2K and lines 207-211.

Line 145: “Using EMS density deep sequencing mapping …” a reference is needed, or a direction to readers to the Methods section, which has the appropriate references.

Response: Thank you for bringing this to our attention. We have added a direction to the methods section, which has relevant references. Please see line 179.

c) Fig. 2D needs a label for the Y-axis

Response: Thanks for bringing it to our attention. Please check that the Y-axis label has been added in Fig. 2D.

d) Fig. 3G, H (and Fig. S3E, F): shouldn’t values for wild-type controls be included to assess the degree of rescue relative to wild-type?

Response: Please refer to Fig. 3C-D and S3A-D, which have been revised to facilitate comparison with the wild-type control.

e) Lines 382-386: there are several references to “Fig. 3.3”, which is not in the manuscript. Do the authors mean Fig. S4? Also, in this section the term “migration” is confusing. I think of migration as the process by which the cell body reaches its appropriate position. It seems like the authors are describing “guidance” or “outgrowth” instead of migration, and in fact, in some cases they use “guidance” and “migration” interchangeably. I suggest sticking to “guidance” or being more specific and calling it “neurite migration”.

Response: Sorry for the confusion. Yes, the figure it referred to is S4. Moreover, as suggested by reviewer 2, we have removed this section to improve the paper’s readability. Please see revised Figure 4.

Line 414: “As one would expect …”. Not sure what’s meant here. Why would one expect that mispositioning the cell body would lead to neurite growth or guidance defects? If there’s a reference demonstrating this, it should be added here.

Response: Sorry for the confusion; we were referring to the positioning of the PLM cell body. This term has been previously described in *Lockhead et al. 2016 (MBoC)* to describe the mispositioning of the PLM cell body posteriorly. Moreover, we should have added references as previous studies suggest that mispositioning of neuronal cell bodies leads to misguidance of tracts, for example, in *unc-53* mutant animals *(Hekimi et al., 1993 (J Neuroscience)).*

However, as described above, please note that the corresponding dataset has been removed.

f) Fig. 4: I think that a representative picture of a “wild-type” animal (really the *shrSi2; lin-15(-)* strain on control RNAi) is needed in 4A to compare to the two conditions shown. Also, the depth coding (which I think is erroneously called “temporal” in the figure legend, which would refer to time) is not useful here: the graded scale is not visible at this resolution, and the path along different depths in wild-type or mutants, and what we should glean from the way it is shown, is not really described.

Response: Please check that the wild-type image has been added and the depth coding removed. Thank you for pointing out the error in the legend. As suggested by Reviewer-2, we are scaling down on this section. Please see Revised Figure 4A.

g) Fig. 5: The *aspm-1* mutation is referred to as a null *(0)* in the figure, but in the text it is referred to as a temperature sensitive *(ts)* mutation. The nomenclature should be consistent.

Response: Please note that for consistency in the text, we have changed it to *(ts)* notation. The mutation is a temperature-sensitive allele; it behaves as a loss-of-function mutation at 25 degrees (as shown in a previous article, Connolly et al., 2014 ([10.1091/mbc.E13-11-0687](https://doi.org/10.1091/mbc.E13-11-0687))).

h) Fig. 6 is more of a summary of the results than an actual model. I envision that a model would suggest a pathway or localization of the studied proteins relative to MTs. I just don’t find this summary very useful, but it doesn’t hurt to have it either.

Response: Thanks for this suggestion. We have revised the figure and caption for the Summary. Please see Figure 7.

In the discussion the authors bring up the actin cytoskeleton, and I found that a bit confusing. For example, in lines 568-572 a possible role for CIT-K in regulating actin dynamics is mentioned, but not elaborated upon, and the next sentence discusses CIT-K function in regulating astral MTs. So, I wasn’t clear as to why the work on actin was mentioned. Similarly, in lines 586-592, the authors seem to bring up the possibility that a CITK-MyosinII-RhoA complex may “bridge” actin and MTs in neurons, as they do in cytokinesis. However, given that the authors did not examine actin in this work (nor do they provide examples of how actin regulates neurite outgrowth from other work), this speculation seems out of place. In the following sentence (lines 592-594) the authors state that they found PLM positioning and guidance defects in the *citk* mutants, but it’s not clear how this fits in a section discussing actin, and/or actin-MT cross-bridging proteins like Kif21b.

Defects found with loss of a cross-bridging protein, like Kif21b, do not provide strong support for a role for actin, as it would be necessary to functionally separate actin vs. MT-regulating

Response: Thanks for the suggestion. We evaluated the dynamics of GFP::UtCH (an actin-binding protein) in PLM neurons of *C. elegans.* We found no observable change in the number of actin trails in *citk-1(0)* animals when compared to wild-type animals (See Figure S3G-H). These observations suggest that, even though *CIT* regulates actin scaffolding in cytokinesis (Bassi et al., 2013; Paolo D Avino, 2017), it does not influence the actin cytoskeleton in the PLM anterior process*.* Please see Figure S3G-H and lines 315-321.

We have revised the discussion accordingly. Please see lines 490-496.

Reviewer #2: The authors use an elegant forward genetic screen to identify modulators of neurite outgrowth linked to microtubule growth in neurons using the advantages of C. elegans. Using a suppressor screen, they identify citk as a regulator of neurite outgrowth through modulation of microtubule growth dynamics/stability. This is a unique role for this protein previously studied primarily as a regulator of cell division previously. The role of citk in neurite extension is further supported by the fact that ASPM, a protein that functions with CITK during cell division, causes similar defects in neurons when mutated. Together, this data argues for roles for these cell division-associated proteins in post-mitotic neurons. Despite my original enthusiasm for this work, there are some missing pieces that are required to provide mechanistic insight into how CITK functions to regulate microtubules and neurite outgrowth. Furthermore, the homology between W02B8.2 and CITK should be confirmed by rescue analysis with a mammalian ortholog. Finally, on a more stylistic point, the writing is drawn out and needs to be more pointed. Long sentences are elegant but, in some cases, the meaning of the sentence is lost. I would encourage the authors to use more succinct language to convey the specific point being made. This is particularly true in the discussion which needs substantial revision. These and other points are discussed below.


1) Section “PLM mechanosensory neurons displayed growth and guidance defects on loss of both citk1 and citk2” can be significantly shortened. Specifically, the discussion of the partially penetrant guidance/positioning phenotypes could be severely abbreviated or eliminated as no follow up mechanistic work was done. It distracts the reader from the main points of the paper if not better integrated.

Response: Thank you for the suggestion. We have removed the section on PLM guidance phenotype. Please see the revised Figure 4

2) Confirmation that W02B8.2 is the worm CITK homolog requires validation. Can the phenotype be suppressed by expression of the mammalian version of CITK or CITN? Both?

Response: We agree this is an interesting experiment. However, we found cloning the mammalian CIT-N RNA from the RNA pool of the mouse cortex challenging due to low transcript abundance (<40 TPM in the cortex of mice). Given that *in-silico* and experimental evidence from functional similarities between *citk-1/citk-2* and *aspm-1* (worm ortholog of the known CIT-binding partner ASPM) strongly support this orthologous relationship, we focus on studying their roles in microtubule regulation in PLM neurons.

3) CIT-N (variant of CITK) has been implicated in Golgi positioning in hippocampal neurons - a dynein-dependent phenotype. Dynein has also been linked to microtubule polarity due to its role in microtubule sliding. Can you expound on the citk loss of function and include a discussion on how the phenotypes could be similar/different to the work on CIT-N in hippocampal neurons? Do dynein hypo- or hyper-morphs give you a similar defect in neuritis length and microtubule polarity? This point should be addressed either with further discussion or experiments as this phenotype has been clearly shown in post-mitotic neurons previously.

Response: Thank you for this interesting suggestion. As rightly pointed out, dynein regulates Golgi positioning and microtubules in axonal and dendritic compartments by minus-end directed motor activity and MT sliding. Interestingly, it has also been shown to remove minus-end-out microtubules from the axonal compartment (Zheng et al., 2008 (Nat Cell Biol) and Castillo et al., 2015 (eLife)). Therefore, we tested the microtubule phenotype in *dhc-1(ts)* mutant animals. As shown in the following figure, *dhc-1(ts)* mutant animals grown at 25°C (non-permissive temperature) showed no change in microtubule polarity in the axon-like anterior process of PLM neuron.

Please check we have furthered the discussion on CIT phenotypes in hippocampal neurons and *citk-1* and *citk-2* in PLM neurons in lines 482-499.

4) As you mention, CIT-K regulates actin dynamics in neurons. I think it is important to discern whether the effect you see is due to microtubules directly or through actin regulation. It would be useful to look at actin dynamics with live imaging or even actin structure to see if there is any reason to think the microtubule defects you see are secondary to actin disruption.

Response: Thank you for pointing this out. We examined actin dynamics in the PLM neurons of *citk-1(0)* animals using GFP::UtCH as previously described (Hegde et al., 2024). However, we did not observe any change in actin dynamics in the anterior process of PLM neurons in *citk-1(0)* animals. Please see Figure S3G-H and lines 315-321.

We have revised the discussion accordingly. Please see lines 490-496

Additionally, a more detailed assessment of the direct nature of the microtubule modifications is necessary in the discussion. This would be aided by a detailed assessment of the cellular localization of citk in these neurons. Does it localize to microtubule plus ends in neurites?

Response: Thank you for this suggestion.

In both *citk-1(0)* and *aspm-1(ts)* mutant animals, we saw perturbation of microtubule plus-end-dynamics (Fig 3A-D and Fig 5B-E) . However, the minus-end puncta (PTRN-1GFP) were unaffected in both *citk-1* and *aspm-1* mutants (Fig S3E-F and Fig S6C-D).

We examined the localization of fluorescently tagged CITK-1 and ASPM-1 in the PLM mechanosensory neurons of C. elegans. We observed a punctate localization of both CITK-1 and ASPM-1 in the cell body as well as the anterior and posterior processes of PLM neurons. we examined if CITK-1 and ASPM-1 colocalize with microtubule plus-end-binding protein EBP-2 in PLM neurons. We found that both GFP::CITK-1 and GFP::ASPM-1 show a significant colocalization with the plus-end-binding protein EBP-2::mCherry. Previously, it has been shown that CITK and ASPM colocalize at the midbody region (Paramasivam et al., 2007 (Cell Cycle) as well as spindle poles in dividing cells. Interestingly, the midbody region has interdigitating plus ends of tightly packed antiparallel microtubules.

Moreover, we examined the localization of fluorescently tagged CITK-1 and ASPM-1 in each other’s mutants. We did not observe any significant change in the localization pattern of ASPM-1 or CITK-1 in the mutant animals. This suggests that their localization is independent of each other in neurons in contrast to cell-division, where ASPM-1 is driving CITK localization to spindle poles (Gai et al., 2016 (EMBO Reports).

From these observations, we conclude that CITK and ASPM-1 regulate microtubule plus-end dynamics as part of the same genetic unit, and both localize at the microtubule plus ends; maybe their MT associating pattern is different.

Please look at Figure 6 and lines 437-458.

5) ASPM mutation gives the same phenotype as CITK, but it is not delineated if this is because it is in parallel or the same cellular pathway. Is the CITK binding domain in ASPM known (or vice versa)? Could this be used in rescue analysis to further determine if these two are functioning together in neurite outgrowth?

ASPM localizes to microtubule plus ends and recruits CITK during cell division. Is plus end localization of CITK lost in ASPM mutants or important for CITK function?

Alternatively, do partial or full loss of ASPM and CITK together produce an additive phenotype?

Response: Thank you for these important suggestions. As we address reviewer-1’s point, we observed no synergistic increase in the microtubule dynamics in the *aspm-1(ts); citk-1(0)* and *aspm-1(ts); citk-2(0)* double mutant animals. This analysis suggests that ASPM and CITK are epistatic in the regulation of the number of growing microtubules in PLM neurons. Please see Fig. 5B-E and S5A-G, lines 405-409.

Furthermore, it has been shown previously that the ASPM C-terminal region coimmunoprecipitated with different myc-tagged CITK fusion proteins, including kinase‐active (FL) or kinase‐dead (KD) full‐length CITK, citron‐N (CN), or the N-terminal or C-terminal half of Citron kinase protein Gai et al., 2017). This suggested that ASPM can directly or indirectly interact with different regions of the CIT protein (Gai et al., 2017). Furthermore, it has been suggested that the N-terminus of the ASPM protein localizes to centrosomes and spindle poles, and the C-terminus tagged with GFP localizes at the midbody region (Paramasivam et al., 2007, Cell Cycle). Please see discussion line

As discussed in the previous section, we found ASPM-1 and CITK-1 colocalization pattern in PLM neurons is mutually independent (See Figure 6, line 450-455, in contrast to cell division, where ASPM-1 is driving CITK localization to spindle poles (Gai et al., 2017).

6) The writing could be made a bit more straightforward. The long/wandering nature of the text makes it a bit difficult to follow. This is true in the discussion most clearly. For example, the paragraph that starts at line 555 is discussion the axon phenotype similarities to previous reports. Then branches into how citk may regulate actin for 1 sentence then astral microtubules in the next without linking these ideas at all. The following paragraph is 2 sentences, with the first one stretching 5 lines. It is unclear what is gained from this paragraph.

Response: Thanks for the suggestion. We have revised the text to make it precise and straightforward.

7) The discussion is a list of things that “should be tested” but weren’t in this study. Some things, particularly the actin cytoskeleton, should be addressed so more definitive conclusions can be drawn. The discussion makes it sound like this is just a replication of previous work without any progress. I don’t think this is the case. A stronger discussion of what has been learned/advanced is needed.

Response: We have made the suggested changes in the discussion. Please check.

**Reviewer #3:** In this manuscript, Sharma et al., studied the function of CITK proteins and ASPM-1 in regulating microtubule dynamics and polarity in C. elegans neurites. They took advantage of the genetic system and identified citk-1 mutations from a genetic screen that is designed to isolate MT regulators. Then they demonstrated that citk-1 and citk-2 functions redundantly in regulating MT dynamics and polarity in the PLM neurons. Because of previous literature linking these two proteins to ASPM1, they tested aspm1’s involvement and found that it also functions in PLM to regulate MTs. The experiments are well designed and executed. The findings are novel and interesting, but lack mechanisms. I support the publication of a revised manuscript. My main suggestions are two-fold: one is on writing and one is on the timing of the function of these proteins.

Major points.
1. The introduction does not reflect an updated set of papers regarding MT organization in neurons. I would suggest the authors to systematically cite papers and separate them into the invertebrate and vertebrate systems because the MT polarity in neurites are somewhat different, and the invertebrate systems often include in vivo analyses.

Response: Please check that we have added relevant references in the introduction. Please see lines 72-80.

2. Despite of the very interesting observations on MT dynamics and polarity phenotypes, the mechanisms of these defects are unclear. I am not suggesting that the authors need to provide a thorough mechanism before this paper can be published. However, it might be very informative to know whether the function of citk and aspm1 are mitotic or postmitotic. In other words, are all the phenotypes caused by defects in early developmental stages (like during mitosis that generates PLM) or are these genes required for the maintenance of the MT dynamics and polarity. Since aspm1 is a temperature allele, that experiment can be easily performed. Are there ways to generate conditional alleles of citk-1 or -2 to test the requirement of these genes for the maintenance of MT properties?

Response: We tested the developmental role of *aspm-1* in the regulation of microtubule dynamics by testing the microtubule dynamics in animals subjected to non-permissive temperature from the L1 growth stage, where the PLM neuron has already differentiated (Hilliard et al., 2006 (Dev Cell)). The mutant animals still show defects in microtubule dynamics equivalent to animals reared at 25 degrees, suggesting that these genes are required for the maintenance of MT dynamics and polarity post-mitotically. Please see Figure S6, lines 410-418.

Moreover, the microtubule dynamics in both *citk-1(0)* and *aspm-1(ts)* mutant animals could be rescued by using the *mec-4* promoter. Mec-4 is a mechanosensory neuron-specific promoter that is activated by a heterodimer of transcription factors, *mec-3* and *unc-86* (Mitani et al., 1993, and Dougan et al., 1998). The *mec-4* promoter is expressed in post-mitotic, terminally differentiated PLM neurons (Hobert et al., 2011, and Hobert et al., 2016). These observations also suggest that these proteins have post-mitotic roles in microtubule dynamics.

Minor points.
3. Line 60 “y-TURC” should be “g-TuRC”.

Response: Thanks for the suggestion. We have modified it. Please see line 82.

4. The Y-axis of Figure 2D is not labeled.

Response: Thanks for pointing that out. We have labeled the axis. Please see the revised Figure 2D.

5. In Figure 1, it seems strange to use mild and strong extension as a measure instead of directly measuring the length of the posterior process.

Response: Thank you, that is a good observation. However, we qualitatively screened the F1s to increase the pace of screening, as 12,422 F1s were to be screened, and measuring the lengths would not have been ideal for the same.

6. It will help to comment on the nature of the b- and a-tubulin mutations in the suppressors. The stop codon mutations are loss of function. Can the authors comment on why the loss of tubulin does not lead to growth defects in the anterior process.

Response: Thank you for asking. We have revised the figure legend of Figure S1 to include the nature of these mutations.

Moreover, previous studies have shown that the loss of tubulin mutations leads to a short neurite defect in the anterior process of ALM and PLM neurons as well. (Savage et al., 1993; Zhang et al., 2017, and Puri et al., 2021). We have not delved into that in this study.

7. Line 294, “… PLM neurons of citk mutant animals seemed to be significantly
295 higher than the number of plus-end-out comets implying a trend…”
Change the statement to a more formal one.

Response: Thank you for your suggestion. Please note that the text has been revised, see lines 266-269.

8. Line414. “…Figure 4E-G)…” There is no 4G in the figure.

Response: We have corrected the text. Thank you for noticing.

**Have all data underlying the figures and results presented in the manuscript been provided?**
Large-scale datasets should be made available via a public repository as described in the *PLOS Genetics* [data availability policy](http://track.editorialmanager.com/CL0/http:%2F%2Fjournals.plos.org%2Fplosgenetics%2Fs%2Fdata-availability/1/010f019768fb1428-43e0e49d-8799-444f-b4eb-0e2731bdbfdb-000000/96az9q541SYp_ns5NxwsNgo15fg8Gbqb_sGyO7IlcgI=215), and numerical data that underlies graphs or summary statistics should be provided in spreadsheet form as supporting information.

Reviewer #1: Yes

Reviewer #2: None

Reviewer #3: Yes

PLOS authors have the option to publish the peer review history of their article ([what does this mean?](https://track.editorialmanager.com/CL0/https:%2F%2Fjournals.plos.org%2Fplosgenetics%2Fs%2Feditorial-and-peer-review-process%23loc-peer-review-history/1/010f019768fb1428-43e0e49d-8799-444f-b4eb-0e2731bdbfdb-000000/bQQKslwkXXXoP2wsGLgALyeSp_9IU3_1g7gI20DCCds=215)). If published, this will include your full peer review and any attached files.


If you choose “no”, your identity will remain anonymous but your review may still be made public.


**Do you want your identity to be public for this peer review?** For information about this choice, including consent withdrawal, please see our [Privacy Policy](https://track.editorialmanager.com/CL0/https:%2F%2Fwww.plos.org%2Fprivacy-policy/1/010f019768fb1428-43e0e49d-8799-444f-b4eb-0e2731bdbfdb-000000/yh_oHX_SvUPEoOzXAQ7UpThpfMhDXoHLHD57BF_hhMw=215).

Reviewer #1: No

Reviewer #2: No

Reviewer #3: No
