# Supplementary material for: Worm orthologues of cytokinesis-associated proteins CIT and ASPM regulate neuronal microtubule dynamics and polarity in C. elegans
Source: PLoS Genet. 2026 Apr 15;22(4):e1012106. doi: 10.1371/journal.pgen.1012106 (PMC13108882; doi:10.1371/journal.pgen.1012106)
Supplement: Attachment — Submitted filename: Response to Reviewers.docx [file pgen.1012106.s020.docx]

PGENETICS-D-25-00512R1
Worm orthologues of cytokinesis-associated proteins CIT and ASPM regulate neuronal microtubule dynamics and polarity in C. elegans
PLOS Genetics

Dear Dr. Ghosh-Roy,

Thank you for submitting your manuscript to PLOS Genetics. After careful consideration, we feel that it has merit but does not fully meet PLOS Genetics's publication criteria as it currently stands. Therefore, we invite you to submit a revised version of the manuscript that addresses the minor points raised during the review process. 

Please submit your revised manuscript within by Apr 02 2026 11:59PM. If you will need more time than this to complete your revisions, please reply to this message or contact the journal office at [plosgenetics@plos.org](mailto:plosgenetics@plos.org). When you're ready to submit your revision, log on to <https://www.editorialmanager.com/pgenetics/> and select the 'Submissions Needing Revision' folder to locate your manuscript file.

Please include the following items when submitting your revised manuscript:

* A letter that responds to each point raised by the editor and reviewer(s). You should upload this letter as a separate file labeled 'Response to Reviewers'. This file does not need to include responses to formatting updates and technical items listed in the 'Journal Requirements' section below.
* A marked-up copy of your manuscript that highlights changes made to the original version. You should upload this as a separate file labeled 'Revised Manuscript with Track Changes'.
* An unmarked version of your revised paper without tracked changes. You should upload this as a separate file labeled 'Manuscript'.

If you would like to make changes to your financial disclosure, competing interests statement, or data availability statement, please make these updates within the submission form at the time of resubmission. Guidelines for resubmitting your figure files are available below the reviewer comments at the end of this letter.

We look forward to receiving your revised manuscript.

Kind regards,

Jeremy Nance
Academic Editor
PLOS Genetics

Pablo Wappner
Section Editor
PLOS Genetics

Aimée Dudley
Editor-in-Chief
PLOS Genetics

Anne Goriely
Editor-in-Chief
PLOS Genetics


**Reviewers' comments:**

Reviewer's Responses to Questions

**Comments to the Authors:
Please note here if the review is uploaded as an attachment.**

Response: We thank the reviewers for their positive comments and suggestions. We appreciate the time and effort dedicated to improving the clarity and technical accuracy of our manuscript.

Reviewer #1: The authors have revised the manuscript by conducting new experiments to address some of the major issues that were brought up in the initial round of review. In particular, the genetic experiments provide support for the hypothesis that ASPM-1 and CITK-1/2 function in the same pathway; a model that was presented in the previous version but not examined experimentally. Moreover, clarification in the writing and the way figures are presented have made the manuscript easier to follow and more compelling. I do still have some minor suggested revisions outlined below, but otherwise feel that the work is acceptable for publication.

a) Line 107: “in the microtubule dynamics may lead to distinct morphological changes…”. I suggest removing the word “may”. These phenotypes are well-established, and you show examples in this paper. So, we know for sure that disruption of MT dynamics cause morphological changes.
Response: Thank you for this suggestion. We agree with reviewer’s assessment that these phenotypes are well established in literature and have removed the word ‘may’. Please see the line 107.

b) Lines 132-133: “in-situ hybridization experiments, …, showed localization of CITK protein in a specific neuronal population in the cortex and thalamus …”. In situ experiments show mRNA localization, not protein. I would reword to say “showed CITK expression in a specific …”.
Response: We thank the reviewer for pointing out this technical error. We agree that the in-situ hybridization (ISH) shows *mRNA* localization. We have updated the text to correctly reflect gene expression rather than protein localization. Please see the lines 132-133, we have rephrased the sentence as suggested.

c) Line 143: The sentence starting “Live imaging of EBP-2::GFP reporter …”. It is not clear here that the authors are starting to describe their own data. I would start this sentence instead saying “Here, using live imaging of an EBP-2::GFP reporter …”. Or maybe put a summarizing sentence before starting to describe the data. Something like “Here we show that the C. elegans orthologs of vertebrate of the CIT kinase, also regulate MT dynamics …”. This would make it clear that your shifting from background information to summarizing what you have discovered.
Response: We appreciate this suggested change. We have incorporated the phrase, “Here, using live imaging of an EBP-2::GFP reporter….”, to make the transition to our data more distinct. Please see the revised line 142-143.

d) Lines 400-402: The sentence “Notably, the fraction of minus-end-out microtubules in the posterior process increases significantly, mirroring the effects of citk-1 overexpression in wild-type animals (Fig S5J)” should be re-written so that the call to the supplemental figure is in the right location, and the authors should also call-out the data in wild-type. So, change to “Notably, the fraction of minus-end-out microtubules in the posterior process increases significantly (Fig S5J), mirroring the effects of citk-1 overexpression in wild-type animals (Fig 3D)”.

Response: We agree that the original citation placement was unclear. We have incorporated the suggested changes to make the call-outs to both new data and data in WT more specific. Please see lines 400-402.

e) Line 408: “suggesting an epistatic genetic relationship”. I don’t think this is the correct term to use. Epistasis implies that the mutants have different phenotypes, and that in the double mutant one phenotype “covers-up” (epistasis literally means “stands upon”) the other. This is most evident when one mutation suppresses the phenotype of the other. What you are discovering here is that combining two mutations does not make the phenotype worse, so I would just say that the results are consistent with the genes functioning in the same pathway.

Response: We agree that ‘epistatic genetic relationship’ is not the most precise term to describe the genetic relationship of *aspm-1* and *citk-1.* Therefore, we have updated the text suggesting that these genes function in the same genetic pathway. Please see line 407-409.

f) Line 414: “equivalent to animals reared at the non-permissive temperature”. I find this confusing. if the mutation is embryonic lethal, how were the authors rearing the animals at the non-permissive temperature continuously? For the continuous non-permissive conditions, were eggs laid at the permissive temperature then shifted? They need to provide more details here, and in the Methods as to how these animals were maintained and when the temperature shifts were done in all their experiments with aspm-1(ts). The description in the methods (“grown at non-permissive temperature of 25°C according to the experimental paradigm”) is too vague.

Response: We apologize for the lack of clarity in the text regarding these shifts to non-permissive temperature. We have now updated the Methods section to explicitly detail the two experimental paradigms used to study the *aspm-1(ts)* allele while bypassing embryonic lethality. We have also ensured that these paradigms are clearly cross-referenced in results section with Figure S6A, which provides a visual timeline of the shifts.

Revised text in methods (line 581-589):

For all experimental purposes, worms were maintained at 20°C, with the exception of the temperature-sensitive(ts) *aspm-1* strain (104). For this strain P0 parents were shifted to non-permissive temperature (25°C) at the L4 stage, and their F1 progeny were imaged at the L4 stage. To investigate the post-mitotic role of ASPM-1 in microtubule regulation, L1 larvae were shifted to 25°C and imaged at the L4 stage, thereby bypassing the embryonic loss of ASPM-1 in differentiating PLM neurons. These two paradigms were distinguished as Growth Condition-I and Growth Condition-II, as illustrated in Fig S6A. All experimental controls were also subjected to identical temperature shifts.

Please also see revised text in results section (line 410-419).

Reviewer #2: This revision has taken into account most of my concerns. I am still a bit worried about the strength of the assertion that these genes are the kinase-less orthologs of the mammalian gene without rescue showing functional redundancy. While cloning the gene may be difficult there are many mammalian orthologs already cloned by other groups. However, at this point, this concern can be remedied by tempering the language a bit. For example, on line 212, I would suggest changing the word "confirmed" which is very strong given the information presented to "suggests" or "argues" or "supports".

Response: We agree with the reviewer’s point that functional rescue with the mammalian ortholog would provide the strongest evidence of orthology. As we have not yet performed these experiments, we have updated the text accordingly at line 212 and throughout the manuscript, referring to *citk-1* as the “suggested kinase-less ortholog of mammalian CIT” to reflect this nuance. Please see lines 121, 141, 197, 212, 238, 461 and 479.

Reviewer #4: The authors have addressed all of the major points raised by the reviewers, and I support publication.

Response: We thank the reviewer for their thoughtful feedback and for supporting the publication of our work.

**Have all data underlying the figures and results presented in the manuscript been provided?**
Large-scale datasets should be made available via a public repository as described in the *PLOS Genetics* [data availability policy](http://track.editorialmanager.com/CL0/http:%2F%2Fjournals.plos.org%2Fplosgenetics%2Fs%2Fdata-availability/1/010f019cb552db71-78fc203f-90bd-4233-a769-1b9da7b6653b-000000/sruIPfPR06Bnoo3FO-z5Y73OV6LxDLTbmhtgfOAMQkw=252), and numerical data that underlies graphs or summary statistics should be provided in spreadsheet form as supporting information.

Reviewer #1: Yes

Reviewer #2: None

Reviewer #4: Yes

PLOS authors have the option to publish the peer review history of their article ([what does this mean?](https://track.editorialmanager.com/CL0/https:%2F%2Fjournals.plos.org%2Fplosgenetics%2Fs%2Feditorial-and-peer-review-process%23loc-peer-review-history/1/010f019cb552db71-78fc203f-90bd-4233-a769-1b9da7b6653b-000000/-4_qaML01c1SQR0y6H4DWC-4TvcCtvxE0TqPf8oPVJY=252)). If published, this will include your full peer review and any attached files.


If you choose “no”, your identity will remain anonymous but your review may still be made public.


**Do you want your identity to be public for this peer review?** For information about this choice, including consent withdrawal, please see our [Privacy Policy](https://track.editorialmanager.com/CL0/https:%2F%2Fwww.plos.org%2Fprivacy-policy/1/010f019cb552db71-78fc203f-90bd-4233-a769-1b9da7b6653b-000000/rZ35lSFi_7MHSgFFwXuuJ7nlW7XaTc_2q5TUHPYTOo0=252).

Reviewer #1: No

Reviewer #2: No

Reviewer #4: No

[NOTE: If reviewer comments were submitted as an attachment file, they will be attached to this email and accessible via the submission site. Please log into your account, locate the manuscript record, and check for the action link "View Attachments". If this link does not appear, there are no attachment files.]

**Figure resubmission:**

While revising your submission, we strongly recommend that you use PLOS’s NAAS tool (<https://ngplosjournals.pagemajik.ai/artanalysis>) to test your figure files. NAAS can convert your figure files to the TIFF file type and meet basic requirements (such as print size, resolution), or provide you with a report on issues that do not meet our requirements and that NAAS cannot fix.

After uploading your figures to PLOS’s NAAS tool - <https://ngplosjournals.pagemajik.ai/artanalysis>, NAAS will process the files provided and display the results in the "Uploaded Files" section of the page as the processing is complete. If the uploaded figures meet our requirements (or NAAS is able to fix the files to meet our requirements), the figure will be marked as "fixed" above. If NAAS is unable to fix the files, a red "failed" label will appear above. When NAAS has confirmed that the figure files meet our requirements, please download the file via the download option, and include these NAAS processed figure files when submitting your revised manuscript.

**Reproducibility:**

To enhance the reproducibility of your results, we recommend that authors deposit laboratory protocols in [protocols.io](http://protocols.io/), where a protocol can be assigned its own identifier (DOI) such that it can be cited independently in the future. Additionally, PLOS ONE offers an option to publish peer-reviewed clinical study protocols. Read more information on sharing protocols at <https://plos.org/protocols?utm_medium=editorial-email&utm_source=authorletters&utm_campaign=protocols>
